# Physiology, ecology and industrial applications of aroma formation in yeast

**DOI:** 10.1093/femsre/fux031

**Published:** 2017-08-17

**Authors:** Maria C Dzialo, Rahel Park, Jan Steensels, Bart Lievens, Kevin J Verstrepen

**Affiliations:** 1 Laboratory for Genetics and Genomics, Centre of Microbial and Plant Genetics (CMPG), KU Leuven, Gaston Geenslaan 1, B-3001 Leuven, Belgium; 2 Laboratory for Systems Biology, VIB Center for Microbiology, Bio-Incubator, Gaston Geenslaan 1, 3001 Leuven, Belgium; 3 Laboratory for Process Microbial Ecology and Bioinspirational Management (PME&BIM), Department of Microbial and Molecular Systems, KU Leuven, Campus De Nayer, Fortsesteenweg 30A B-2860 Sint-Katelijne Waver, Belgium

## Abstract

Yeast cells are often employed in industrial fermentation processes for their ability to efficiently convert relatively high concentrations of sugars into ethanol and carbon dioxide. Additionally, fermenting yeast cells produce a wide range of other compounds, including various higher alcohols, carbonyl compounds, phenolic compounds, fatty acid derivatives and sulfur compounds. Interestingly, many of these secondary metabolites are volatile and have pungent aromas that are often vital for product quality. In this review, we summarize the different biochemical pathways underlying aroma production in yeast as well as the relevance of these compounds for industrial applications and the factors that influence their production during fermentation. Additionally, we discuss the different physiological and ecological roles of aroma-active metabolites, including recent findings that point at their role as signaling molecules and attractants for insect vectors.

## Teaser

This review explores the biochemical pathways leading to production of a wide array of aroma compounds, the various industrial applications that have been developed around use of aroma compounds, as well as the newly uncovered physiological and ecological roles the various compounds may play.

## INTRODUCTION

When presented with the appropriate nutrients, yeasts produce complex bouquets of aroma compounds including esters, higher alcohols, carbonyls, fatty acid derivatives and sulfur compounds. Moreover, while not directly synthesized by yeasts, volatile thiols and monoterpenes are sometimes released from odorless precursors by yeast-derived enzymes (Tominaga *et al.*^1998^; Moreira *et al.*^2005^). Our understanding of the fermentation process and the associated aroma production by yeast has increased exponentially over the last centuries, from the discovery of yeast cells in 1680, to the sequencing of the entire *Saccharomyces cerevisiae* genome just two decades ago (Goffeau *et al.*^1996^), and capping off with an in-depth look at the phenotypic and genetic diversity of nearly 200 industrial yeasts last year, including a detailed profiling of differences in aroma formation (Gallone *et al.*^2016^; Gonçalves *et al.*^2016^). Interestingly, these recent studies demonstrate that humans have helped drive the domestication of yeasts, at least partly based on their ability to selectively produce desired aromas and reduce unwanted compounds.

Given its importance in product quality, much effort has been devoted to fine-tune flavor production by yeast in an industrial setting. Globally, two approaches can be applied to steer the yeast's physiology to alter aroma production: adjusting the fermentation environment or modifying the genotype of the production strain. Adjusting the environmental parameters can be a convenient, often very powerful, way to optimize production without complex biotechnological procedures nor a thorough understanding of basic yeast physiology. However, given the recent expansion of the available yeast biodiversity, strategies to modify yeasts and the genetic toolbox to genetically engineer strains, biotechnologists can now select or develop new yeasts with aromatic properties far beyond what is achievable through adjustment of environmental parameters.

While humans have been advancing, and refining the exploitation of yeast aroma for several millennia, it remained unknown why yeast cells produce these flavor-active molecules in the first place. Over the past decades, several hypotheses for possible physiological roles have been proposed, including synthesis of specific cellular building blocks, redox balancing and detoxification reactions, but the evidence for these remained very limited. Recent studies, however, have begun to uncover a fundamental and central role of aroma production in the lifestyle of yeast. Specifically, it has been shown that yeast-derived volatiles can have integral roles in natural environments, ranging from signaling information to animal vectors, regulation of fungal growth and communication between yeast cells or colonies (Richard *et al.*^1996^; Bruce *et al.*^2005^; Leroy *et al.*^2011^; Davis *et al.*^2013^). The interaction between yeasts and insects has been studied intensively the past decade and there is increasing evidence that attraction of many insect species to fermenting fruits is mediated by the volatiles emitted by the yeasts rather than by the fruit itself (Becher *et al.*^2012^).

**Figure 1.**
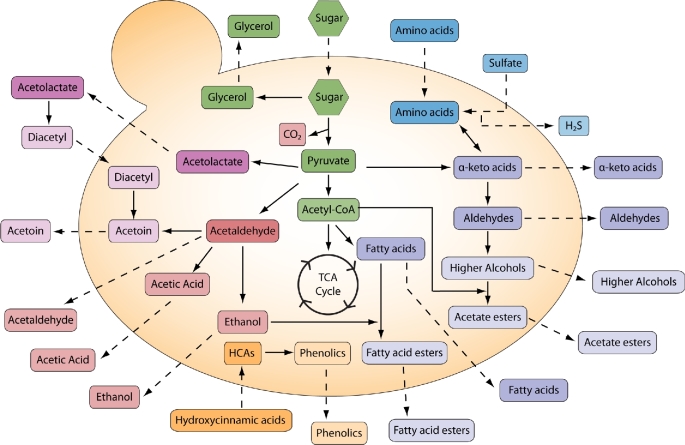
**Overview of aroma compound production**. This review covers a large array of aroma compounds produced during yeast fermentation. The basic fermentation of pyruvate (green/red) leads to several carbon-based compounds, including ethanol and carbon dioxide. Pyruvate also feeds into the anabolism of amino acids, leading to production of vicinal diketones (pink). Metabolism of amino acids is responsible for numerous aroma compounds including higher alcohols and esters (purple) as well as sulfur-containing compounds (blue). Additionally, the phenolic compounds are derived from molecules found in the media (orange). Compounds shown in darker shades are considered intermediates while lighter shades are aroma compounds discussed in this review. Dotted lines indicate import/export of compounds, solid lines represent biochemical reactions (not indicative of number of reactions).

In this review, we provide an overview of the current understanding of aroma production in yeasts in an industrial, physiological and ecological context. We attempt to provide a more global review covering major compounds discussed commonly in industry and ecology (Fig. 1). For each metabolite category, we first illustrate the biochemical pathways which are crucial for understanding the rationale behind much of the industrial research. Note that much of the biochemical review in this paper will refer to *Saccharomyces cerevisiae* since research into the specific mechanisms of the fermentation process is commonly based on this species, given its central role as a model organism and as a robust fermenter in industry. We then discuss the industrial roles of the aroma compounds that humans have developed. We also highlight key environmental parameters, such as temperature and medium composition, that are commonly adjusted to affect specific compound production as well as some modifications to genetic background that have been developed to influence aroma production. Lastly, we explore some of the possible physiological and ecological roles of these aroma compounds.

## PRIMARY FERMENTATION METABOLITES: ETHANOL

In many industrial fermentation processes, ethanol is the most important compound produced by yeast. Moreover, it is the production of this primary metabolite that originally sparked interest for the fermentation of beverages. Early civilizations developed fermentation methods to exploit the benefits of ethanol; ethanol prolongs shelf-life, improves digestibility and acts as a euphoriant (Alba-Lois and Segal-Kischinevzky 2010). Today, ethanol still forms the basis of many fermented products, either destined for consumption or for renewable energy. Moreover, ethanol is a volatile aroma compound, although its sensorial properties are perhaps less pronounced than some of the more flavorful molecules that are also formed as byproducts of the fermentation pathway.

### Biochemistry of ethanol production

Although yeasts have been utilized for their fermentative capacity for millennia, the molecular components of this basic pathway were only discovered in the last few decades (Bennetzen and Hall 1982; Schmitt, Ciriacy and Zimmermann 1983).

**Figure 2.**
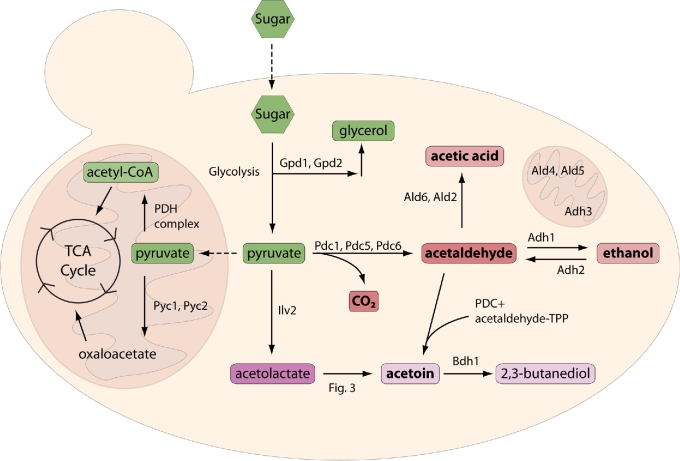
**Production of ethanol, acetaldehyde, acetic acid, and CO_**2**_**. Fermentable carbons are assimilated from the medium and converted to glycerol or pyruvate via glycolysis. Pyruvate can be shuttled towards the TCA cycle and respiration (left) or towards alcoholic fermentation (right). For some conversions, multiple enzymes can perform the reaction and are indicated on the figure. Note: Ald4, Ald5 and Adh3 are mitochondrial enzymes but perform the same reactions as the other cytosolic ALD and ADH enzymes.

Central metabolism begins with the basic conversion of sugars into pyruvate, yielding energy in the form of ATP and reduced NADH cofactors. The divergence of pyruvate after glycolysis is an essential regulatory point in metabolism, which has made it a hotspot for biochemical and industrial research. There are two basic directions pyruvate can take at this point: fermentation or respiration. In most eukaryotes, this is dependent on the presence of oxygen. In aerobic conditions, pyruvate will be converted to acetyl-coA by actions of a pyruvate dehydrogenase and head towards the citric acid cycle (Fig. 2). Under fermentative (anaerobic) conditions, pyruvate is diverted towards fermentation.

Conversion of pyruvate to ethanol is a two-step process. First, pyruvate is converted to acetaldehyde by a pyruvate decarboxylase (PDC), releasing carbon dioxide as waste. There are three confirmed PDC enzymes encoded in the *Saccharomyces cerevisiae* genome (Saccharomyces Genome Database; Cherry *et al.*^2012^). These enzymes act as a key metabolic branch point between fermentation and respiration. In direct competition with pyruvate dehydrogenase, PDCs can remove excess pyruvate from the pathway and divert it towards ethanol production.

Acetaldehyde is subsequently converted into ethanol by an alcohol dehydrogenase (ADH). This type of oxidoreductase can catalyze the reversible interconversion of alcohols and the corresponding aldehydes or ketones. The wide array of substrates available for ADHs throughout the metabolic pathways requires substantial regulation to ensure a balance of the desired products and intermediates. It is therefore not surprising that eukaryotes, even humans, have numerous ADH enzymes. Even a simple eukaryote like *S. cerevisiae* has seven ADH genes as well as several aryl-alcohol dehydrogenases (AAD). Adh1 is the primary enzyme for producing ethanol during fermentation and for replenishing the pool of NAD^+^, while Adh2 is glucose repressible and will oxidize ethanol as a carbon source when needed (Leskovac, Trivić and Pericin 2002). Adh3 is constitutively expressed during both ethanol production and utilization but as it is expressed in the mitochondria, its primary role is likely to maintain redox balance (Bakker *et al.*^2001^; de Smidt, du Preez and Albertyn 2012).

### Ethanol in industry

Ethanol is an important yeast metabolite for most products involving yeast fermentation. It is a vital ingredient of fermented beverages and is used as a prominent renewable biofuel but ethanol also plays a role in product quality of other fermented products where the connection is perhaps more obscure. For example, during baking, ethanol produced by yeast has a strong impact on dough extensibility and gluten agglomeration (Jayaram *et al.*^2014^). During cocoa fermentations, the ethanol produced by yeast serves as a carbon source for acetic acid bacteria (which are vital for cocoa flavor) and triggers biochemical reactions within the cocoa bean that lead to the production of various aromas and aroma precursors (Hansen, del Olmo and Burri 1998).

Given the central role of ethanol in alcoholic fermentation processes, much research has focused on improving speed and efficiency of alcohol production by yeasts over the past few decades, especially in the bioethanol industry. Interestingly, there is also an emerging trend towards fermented beverages with *reduced* ethanol content (Wilkinson and Jiranck 2013; WHO 2014). This is driven by the increasing demand from both consumers and producers to reduce problems associated with high alcohol levels. Too much ethanol can compromise quality of the product and excessive alcohol intake is associated with various health issues. From a financial standpoint, high alcohol content can increase the costs to the consumer in countries where taxes are calculated based on ethanol content.

### Environmental parameters and ethanol production

Modifying the fermentation parameters, including carbon sources, trace elements and even temperature, has proven to be effective measures for altering ethanol production by industrial yeasts (Table 1).

 However, the positive effects of these medium adjustments are often strain dependent (Remize, Sablayrolles and Dequin 2000), and in case of food production, the potentially disadvantageous side effect on aroma must be assessed carefully. Other, more adventurous, strategies have been recently described. For example, ‘electro-fermentation’ imposes an electrical field on the fermentation to serve as an alternative source of reducing and oxidizing power (Schievano *et al.*^2016^). Application of a static potential of up to 15 V (without any resulting current) to a *S. cerevisiae* culture resulted in a 2-fold yield of ethanol (reaching 14% v/v) and 2 to 3-fold faster fermentation rate (Mathew *et al.*^2015^). In another strategy, Lam *et al.* (2014) strengthened the opposing potassium and proton electrochemical membrane gradients during fermentations, which led to an enhanced resistance to multiple alcohols, including ethanol (Lam *et al.*^2014^).

**Table 1.** Effect of environmental parameters on ethanol production.

**Parameter**	**Condition**	**Effect on ethanol production**	**Reference**
**Temperature**	Above optimal	Decrease (lower ethanol tolerance)	Coleman *et al.* (2007)
**pH**	Increase	Increase (increased proton electrochemical gradient)	Lam *et al.* (2014)
**Oxygen**	Increase	Increase (higher cell viability)	Alfenore *et al.* (2004)
**Medium composition**			
***C source***	Preferred sugars (glucose, sucrose)	Decrease (undesired side effects on physiology)	Verstrepen *et al.* (2004)
***N source***	NH_4_, glutamate	Decrease (compared to amino acids)	Albers *et al.* (1996)
***Metal ions***	Supplementation	Increase	Tosun and Ergun (2007)
***Vitamins***	Supplementation	Increase	Alfenore *et al.* (2002)
***Lipids (fatty acids, sterols)***	Supplementation	Increase	Pham *et al.* (2010)
***Nutrient-rich mixtures***	Supplementation	Increase	Jones and Ingledew (1994)
***Potassium***	Supplementation	Increase (increased potassium membrane gradient)	Lam *et al.* (2014)
**Electric field**	Application of 15V	Increase (alternative source of redox power)	Mathew *et al.* (2015)
**Enzyme (Amylase)**	Supplementation	Increase (more available sugars)	Nigam and Singh (1995)

### Genetic factors and ethanol production

One of the easiest ways to obtain yeasts with modulated ethanol production capacity is screening the available natural biodiversity. Most fermentation processes are conducted with *S. cerevisiae*, or very related species, such as *S. pastorianus* (lager beer) or *S. bayanus* (some wines). It has been shown numerous times that traits such as ethanol tolerance or ethanol accumulation capacity are strain dependent within *S. cerevisiae* (Swinnen *et al*. 2012; Snoek *et al*. 2015; Gallone *et al.*^2016^) and nature often harbors superior variants. For example, Brazilian bioethanol plants initially inoculated with baker's yeasts but were rapidly taken over by wild autochthonous strains (Basso *et al.*^2008^). These wild contaminants have been used as commercial starter cultures ever since. Moreover, while *Saccharomyces* spp. are still the preferred organism for most fermentation processes, alternative species such as *Brettanomyces bruxellensis*, *Metschnikowia pulcherrima*, *Torulaspora delbrueckii, Saccharomycodes ludwigii and Zygosaccharomyces rouxii* produce increased (Passoth, Blomqvist and Schnürer 2007; Steensels and Verstrepen 2014; Radecka *et al.*^2015^) or decreased (Contreras *et al.*^2015^; De Francesco *et al.*^2015^; Morales *et al.*^2015^; Canonico *et al.*^2016^) levels of ethanol, thereby further expanding the portfolio of potential industrial yeasts.

Nevertheless, numerous research projects have aimed to modify ethanol production, or fermentation efficiency in general, within a specific strain by altering the genetic background. However, the large number of enzymes and branch points involved can complicate the results of adjusting genes and metabolites involved in central carbon metabolism. Ethanol production of industrial strains has been adjusted by various strategies, including increased ethanol tolerance (Zhao and Bai 2009; Lam *et al.*^2014^; Snoek *et al.*^2015^; Voordeckers *et al.*^2015^; Ohta *et al.*^2016^), reduced production of alternative metabolites (e.g. glycerol) (Remize, Sablayrolles and Dequin 2000; Pagliardini *et al.*^2013^; Hubmann *et al.*2013a) and increased ethanol accumulation capacity (Pais *et al.*^2013^; Snoek *et al.*^2015^).

During many industrial fermentation processes, especially in bioethanol fermentations or high-gravity brewing, yeast encounter extremely high ethanol concentrations, sometimes reaching up to 20%–25% v/v. This can quickly become toxic to the cells and has thus led to considerable efforts in increasing ethanol tolerance of industrial yeast strains. Therefore, many studies target the improvement of ethanol tolerance. Some recent and innovative approaches are highlighted here (see Zhao and Bai 2009; Snoek, Verstrepen and Voordeckers 2016 for a more comprehensive overview). Natural variations in *MKT1* (a nuclease), *SWS2* (a mitochondrial ribosomal protein) and *APJ1* (a chaperone with a role in SUMO-mediated protein degradation), though not traditionally linked to ethanol tolerance, account for the increased ethanol tolerance of the Brazilian bioethanol strain VR1 (Swinnen *et al.*^2012^). Variations in the metabolome, namely accumulation of valine via deletion of *LEU4* and *LEU9* (which encode for key enzymes connecting valine to leucine synthesis) or reduction of inositol levels by deletion of *INM2* (involved in inositol biosynthesis), also effectively increase ethanol tolerance (Ohta *et al.*^2016^). Global transcription machinery engineering, a high-throughput genetic technology, was used to find variants of the global transcription factor Spt1 with increased ethanol tolerance (Alper *et al.*^2006^). The mutated versions of this protein led to widespread transcriptional reprogramming when introduced in yeast, and some of the resulting mutants demonstrated improved ethanol tolerance (Alper *et al.*^2006^). Other high-throughput strategies, such as TALENs (transcription activator-like effector nucleases)-assisted multiplex editing and robot-assisted genome shuffling, have also yielded improvements in strain ethanol tolerance (Snoek *et al.*^2015^; Zhang *et al.*2015c). Long-term evolution has also been demonstrated as an effective measure to increase ethanol tolerance. Turbidostat cultures grown continuously for over 2 years with gradually increasing ethanol concentrations yielded tolerant variants with mutations in *PRT1* (subunit of the eukaryotic translation initiation factor 3), *VPS70* (involved in vacuolar protein sorting) and *MEX67* (poly(A)RNA-binding protein involved in nuclear mRNA export) (Voordeckers *et al.*^2015^).

Modification of glycerol synthesis can also affect ethanol production. During anaerobic growth, glycerol serves as an ‘electron sink’ to re-oxidize NADH generated during biosynthesis and concentrations can reach up to 5 g/L during industrial fermentations (Nielsen *et al.*^2013^). Deletion of glycerol synthesis genes *GPD1* and *GPD2* directly decreases glycerol levels with a resultant increase in ethanol (Nissen *et al.*^2000^). Natural variations of *GPD1, HOT1* (a transcription factor involved in glycerol synthesis), *SSK1* (a phosphorelay protein involved in osmoregulation) and *SMP1* (a transcription factor involved in osmotic stress response) also result in decreased glycerol to ethanol ratios during fermentation (Hubmann *et al*. 2013a,b). Additionally, expression of a non-phosphorylating, NADP^+^-dependent GAP reduces formation of cytosolic NADH and results in decreased glycerol with increased ethanol (Bro *et al.*^2006^).

Lastly, total ethanol accumulation can be improved. This trait is related to ethanol tolerance, but different molecular mechanisms can underlie them (Pais *et al.*^2013^). Reverse metabolic engineering identified three natural alleles that can improve ethanol accumulation capacity in yeast: *ADE1* (a nucleotide synthase), *URA3* (a decarboxylase involved in pyrimidine synthesis) and *KIN3* (kinase involved in ethanol tolerance) (Pais *et al.*^2013^). In another study, large-scale, robot-assisted genome shuffling yielded hybrids with an increased ethanol accumulation of up to 7% relative to a widely applied bioethanol strain (Ethanol Red), but the underlying genetic factors were not identified (Snoek *et al.*^2015^).

Some studies aim to reduce ethanol production to fit growing trends of low alcohol beverages. The main challenge is to achieve the ethanol reduction without the loss of product quality, as ethanol production is often tightly linked to production of other volatile metabolites. Methods for removal of ethanol during or after the fermentation process exist, however, while efficient, current strategies are often costly or carry along undesired side effects, such as inferior aroma (Varela *et al.*^2015^). Newer strategies aim to limit the amount of ethanol produced by the yeast, mainly by altering the central carbon flux or regulating redox balance (Kutyna *et al.*^2010^; Goold *et al.*^2017^). For example, deletion of *PDC1* or *ADH1*, the major ethanol production line, reduces ethanol production (Nevoigt and Stahl 1996; Cordier *et al.*^2007^). Overexpression of glycerol synthesis genes such as *GPD1* and *FPS1* shifts carbon flux away from ethanol and towards glycerol synthesis (Nevoigt and Stahl 1996; Remize, Barnavon and Dequin 2001; Cambon *et al.*^2006^; Cordier *et al.*^2007^).

### Physiological and ecological roles of ethanol

Eukaryotic cells typically opt for respiration when possible as it offers a higher yield of ATP per molecule of glucose. Certain yeasts, including *S. cerevisiae*, opt to ferment even in the presence of oxygen (De Deken 1966). This so-called Crabtree effect is paradoxical, as the energy yield is significantly lower. However, it is believed that the rate of ATP production (amount per time) is actually higher through fermentation, allowing for faster growth. Moreover, ethanol is highly toxic to most other microbes, which may help yeast cells compete with faster-growing competitors (Rozpędowska *et al.*^2011^). Although much of metabolic flux is diverted to ethanol, it is important to note that a fraction of the carbon is still shuttled to the TCA cycle, which forms important aroma precursors through reactions associated with amino acid metabolism.

Ethanol production by fermenting yeast cells may also have an indirect role in ecology. Several studies indicate that ethanol influences the behavior of insects that inhabit the same natural niches. Fruit flies are strongly attracted to rotting fruits due to high concentrations of fermentation products, including ethanol (Becher *et al.*^2012^). In fact, ethanol provides a nuanced signal for preferential oviposition sites among closely related *Drosophila* (Diptera: Drosophilidae) species. Ethanol tolerance of adult flies of different species seems to correlate with preference for ethanol-rich oviposition substrate (Sumethasorn and Turner 2016). *Drosophila melanogaster* is highly ethanol tolerant and in laboratory conditions will lay twice as many eggs on ethanol-rich media than the ethanol-sensitive *D. mauritiana*. Moreover, the same species from differing climates can demonstrate variations in both ethanol tolerance and ovipositioning preference. *Drosophila melanogaster* from temperate populations, such as Europe, has higher ethanol tolerance than populations from Africa (Zhu and Fry 2015) and higher ethanol concentrations increase ovipositioning frequency from the European fly, but reduced frequency from African flies (Sumethasorn and Turner 2016).

The effect of ethanol content on ovipositioning has also been linked to the presence of parasitic wasps. *Drosophila melanogaster* increases egg laying on ethanol-rich substrate when there are parasitic wasps in the vicinity (Kacsoh *et al.*^2013^). Subsequently, eggs laid by the wasps suffer increased mortality if the host ingests ethanol-rich substrates (Milan, Kacsoh and Schlenke 2012) and even dilute levels of ethanol can reduce the total number of parasitoid eggs laid in the larvae. The preference for an ethanol-containing ovipositioning site can strongly depend on the presence of suitable, ethanol-free food sources nearby. When the alternative ethanol-free substrate is close, flies prefer the ethanol-containing substrate. As distance increases, preference for the ethanol rapidly declines (Sumethasorn and Turner 2016). Taken together, this suggests that fruit flies are continuously reevaluating the relative positions of the available substrates, potentially to ensure survival. They seem to prefer harsh (ethanol-rich) environments to protect the eggs and freshly hatched larvae, but only if a suitable, less harsh food source is nearby for the larvae to find.

The use of microbially produced compounds is a relatively recent and recurrent approach currently being used as attractants for various biological pests, and several examples will appear throughout this review. One very recent example of this tactic is the use of ethanol-containing mixtures against the avian parasite *Philornis downsi* (Diptera: Muscidae). This South American-native fly has recently invaded the Galapagos and its larvae have been feeding on the nestlings of the famous Darwin's finches (Kleindorfer and Dudaniec 2016). *Philornis downsi* adults feed on fermented substrates, and ethanol plays a crucial role in guiding them to the food source. When ethanol is mixed with acetic acid, it effectively and specifically attracts *P. downsi* over non-target insects (Cha *et al.*^2016^). Similarly, the combination of ethanol and acetic acid has been suggested as a useful and inexpensive lure for trapping other insects such as pathogen-carrying *Muscina stabulans* (Diptera: Muscidae) and *Fannia canicularis* (Diptera: Muscidae) (Landolt, Cha and Zack 2015), as well as the corn pest *Carpophilus humeralis* (Coleoptera: Nitidulidae) (Nout and Bartelt 1998).

Insects are not the only organisms to be affected by ethanol. Originally thought to be solely soil dwelling, the nematode *Caenorhabditis elegans* is frequently found in rotting fruits, stems and flowers (Félix and Braendle 2010). It is therefore likely that *C. elegans* larvae encounter ethanol from microbial fermentation in its natural environment. While high concentrations of ethanol (above 100 mM) result in slower development, decreased fertility and shorter life span (Davis, Li and Rankin 2008), at lower concentrations, ethanol appears to have beneficial survival effects, prolonging the lifespan of the stress-resistant larval stage (Castro *et al.*^2012^). Since the nematode larvae do not appear to actively seek out ethanol (Patananan *et al.*^2015^), it is hypothesized that the ethanol could provide a temporary carbon source to ensure the larvae survive until proper food sources are found. Interestingly, ethanol can influence *C. elegans* negatively through a complex multispecies interaction: the yeast-produced ethanol can enhance the growth of several *Acinetobacter* species, and in turn make them more efficient to withstand and even kill their natural predator, *C. elegans* (Smith, Des Etages and Snyder 2004).

Certain primates are also attracted to fermenting food. Complex microbial communities in nectar sources produce diverse volatiles that make them more attractive to potential animal pollinators. The nectar of bertam palm (*Eugessona tristis*), a popular food source for several insects and small animals, can contain up to 3.8% ethanol (Wiens *et al.*^2008^). Behavioral studies indicate that these nectar-seeking animals, specifically the primate slow loris (*Nycticebus coucana*) and the lemur aye-aye (*Daubentonia madagascariensis*), preferentially feed on nectar containing ethanol (Gochman, Brown and Dominy 2016). Interestingly, aye-ayes have a mutation in their *ADH4* gene resulting in a 40-fold increase of their ethanol metabolism compared to most of the primates, potentially explaining why they do not get intoxicated on the high-alcohol food (Carrigan *et al.*^2015^).

## PRIMARY FERMENTATION METABOLITES: CO_2_, ACETALDEHYDE AND ACETIC ACID

### Biochemistry of CO_2_, acetaldehyde and acetic acid production

As mentioned, under fermentative (anaerobic) conditions, pyruvate is diverted towards ethanol in a two-step process (Fig. 2). Pyruvate is first converted to acetaldehyde with concomitant release of carbon dioxide (CO_2_) by PDC. The two major PDC enzymes, Pdc1 and Pdc5, are the major contributors to the decarboxylation activity in the cell and therefore directly control levels of acetaldehyde and CO_2_ (Kulak *et al.*^2014^). Pdc6 is primarily utilized during growth on non-fermentable carbon sources (Hohmann 1991). One would expect then that in a *PDC1* deletion the levels of acetaldehyde to significantly drop. However, in certain conditions, deletion of this enzyme demonstrates an increase in acetaldehyde (Curiel *et al.*^2016^). It is hypothesized that Pdc5 can compensate for up to 70% of the required PDC activity, indicating a possible compensatory mechanism to maintain flux towards acetaldehyde and subsequent ethanol production (Wang *et al.*^2015^). Furthermore, Pdc5 has a higher specific activity which may allow it to directly compete with the respiratory pyruvate dehydrogenase and may help push more pyruvate towards ethanol (Agarwal, Uppada and Noronha 2013).

Acetaldehyde can then continue towards ethanol via ADH activity, or it can be acted on by an aldehyde dehydrogenase (ALD) to produce acetic acid. Like the ADHs, there are several ALDs, further expanding the level of regulation centered around carbon flux. If acetaldehyde is produced cytosolically, it can be acted on by Ald6 or Ald2; if produced in the mitochondria, it is converted by Ald4 or Ald5. Additionally, an acetaldehyde molecule still covalently linked to the PDC complex (via the bound thiamine pyrophosphate) can interact with an additional acetaldehyde to form acetoin (Fig. 2).

### Carbon dioxide in industry

While humans do not typically associate an odor with carbon dioxide, its production is important in some industrial processes and is detectable by other organisms (see Physiological roles of CO_2_). CO_2_ is responsible for the natural carbonation of fermented beverages and adequate gas production is arguably the most important selection criterion for commercial baker's yeasts, as proper leavening requires rapid and sufficient CO_2_ release (Randez-Gil, Córcoles-Sáez and Prieto 2013). Therefore, most optimization for increased speed of CO_2_ production has been performed in bread yeasts.

### Environmental parameters and CO_2_ production

Most bread fermentations should only take 1–2 h which requires a quick onset of the fermentation process to rapidly and effectively produce large volumes of CO_2_. To this end, various dough parameters can be adjusted to speed up CO_2_ production (Table 2). Optimization of the physiological state of the yeasts before introducing them into the dough can drastically improve leavening ability. This can be accomplished by pre-soaking and thus reactivating dry yeast prior to starting the bread fermentation (Gelinas 2010). Additionally, adjusting the way that the dried yeasts are produced, for example, by optimizing the medium in which they are grown, the timing at which the yeast cells are harvested, or the specific drying protocol, can increase yeast viability and vitality during bread fermentations (Galdieri *et al.*^2010^; Rezaei *et al.*^2014^).

**Table 2.** Effect of environmental parameters on CO_2_ production.

**Parameter**	**Condition**	**Effect on CO_**2**_ production**	**Reference**
**Temperature**	Decrease storage T of yeasted dough	Decrease	Sasano *et al.* (2012)
**Dough mixing time**	Increase	Increase	Sahlström *et al.* (2004)
**Medium composition**			
***C source availability***	Increase	Increase, however, risk for osmotic stress	Sahlström *et al.* (2004)
***Salt***	Increase	Decrease (stress), however, better CO_2_ containment	Lynch *et al.* (2009), Toyosaki and Sakane (2013)
***Nutrient mixes***			
***(wheat bran)***	Supplementation	Increase	Hemdane *et al.* (2016)
**Enzyme (amylase)**	Supplementation	Increase (more available sugars)	Struyf *et al.* (2017)

### Genetic factors and CO_2_ production

In general, the ability to ferment specific bread-associated sugars (namely maltose, glucose, sucrose, and fructose) has been altered to improve CO_2_ production, or the leavening ability, of baker's yeast. One of the most common problems associated with dough fermentation is the considerable lag between fermentation of preferred sugars, glucose and sucrose, and fermentation of maltose, the principle fermentable sugar in bread dough. Catabolite repression slows down the switch and subsequently lengthens leavening time (Gancedo 1998). Therefore, genes associated with glucose repression and maltose utilization have often been strategically targeted for genetic modification (Osinga *et al.*^1989^; Sun *et al.*^2012^; Lin *et al*. 2014, 2015b; Zhang *et al*. 2015a,b). Alternatively, maltose utilization can be improved by selecting mutants on medium containing fermentable maltose with non-metabolizable glucose analogs. Such strategies yield strains with deficiencies in catabolite repression that could co-consume glucose and maltose resulting in faster dough leavening (Randez-Gil and Sanz 1994; Rincón *et al.*^2001^; Salema-Oom *et al.*^2011^). Similar mutants could potentially reduce the lag time in the beer brewing fermentations as well (New *et al.*^2014^). Consecutive rounds of mass mating and selection have also yielded commercial strains with improved maltose utilization (Higgins *et al.*^2001^).

Yeast encounter various severe stresses during bread fermentations, such as high sugar and salt concentrations, which reduces their performance (Aslankoohi *et al.*^2013^). Improvements of general stress resistance of industrial yeast have been shown to yield faster bread fermentations. This is generally achieved by increasing production of glycerol and other small protective molecules such as proline and trehalose (Shima and Takagi 2009). Overexpression of glycerol synthesis genes, such as *GPD1*, increases glycerol accumulation and subsequent osmotolerance (Aslankoohi *et al.*^2015^). Modification of proline permeases (*PUT4*) or proline biosynthesis genes (*PRO1*) increases proline accumulation and improves osmo-, cryo- and halotolerance (Kaino *et al.*^2008^; Poole *et al.*^2009^; Sasano *et al.*^2012^). Disruption of trehalose degradation (*NTH1*, *ATH1*) or efflux (*FPS1*) increases intracellular trehalose levels and improves freeze tolerance (Shima *et al.*^1999^; Izawa *et al.*^2004^; Sasano *et al.*^2012^; Sun *et al.*^2016^). Overexpression of *CAF16* and *ORC6*, two genes that are upregulated during osmotic and cryostress, also improves overall stress tolerance of the yeast during baking (Pérez-Torrado *et al.*^2010^). Directed evolution has also been used to improve stress tolerance in baker's yeast. Ultraviolet mutagenesis followed by 200 consecutive freeze–thaw cycles yielded mutants with improved freeze tolerance, without undesirable side effects in other fermentation properties (Teunissen *et al.*^2002^).

### Acetaldehyde and acetic acid in industry

Acetaldehyde is the central intermediate between pyruvate and ethanol but it is also an important aroma compound. It is quantitatively the most abundant aldehyde in most fermented products including apple juice and spirits (Miyake and Shibamoto 1993), beer (Margalith 1981; Adams and Moss 1995), cider and perry (Williams 1975), wine (Liu and Pilone 2000), cheese (Engels *et al.*^1997^), yoghurt (Zourari, Accolas and Desmazeaud 1992) and ripened butter (Lindsay, Day and Sandine 1965). Production of acetaldehyde has direct influence on the final product's aroma, levels of ethanol production, as well as product stability and toxicology (Romano *et al.*^1994^). At low levels, acetaldehyde provides a pleasant, fruity aroma and is a decisive aromatic compound of many sherry-type and port wines (Zea *et al.*^2015^). However, it is also notorious for its undesirable green apple-like or grassy flavor when exceeding threshold levels. This threshold varies drastically between matrices, with 10 μg/g (ppm) reported for beer (Meilgaard 1982), 30 μg/g for cider (Williams 1974) and up to 130 μg/g for certain wines (Berg *et al.*^1955^). Chemical conversions during aging can also increase overall acetaldehyde concentrations of fermented beverages over time (Vanderhaegen *et al.*^2003^).

Apart from its direct effect on flavor, acetaldehyde arguably has even a more important role indirectly. The molecule is extremely reactive and can react with various other compounds. In red wines, for example, acetaldehyde influences various parameters not directly linked to aroma. It can bind sulfur dioxide (SO_2_), which drastically reduces the effectiveness of this antimicrobial agent, thereby facilitating spoilage (Liu and Pilone 2000). Acetaldehyde can also react with tannins, which are naturally occurring polyphenols in grapes, to form irreversible, covalent bridges, resulting in a reduction of the dry, puckering mouthfeel (‘astringency’) that is associated with these compounds (Mercurio and Smith 2008). A similar condensation reaction between anthocyanins or between anthocyanins and tannins mediated by acetaldehyde-bridged complexes is observed, resulting in polymeric pigments that influence wine color. These highly stable complexes are not susceptible to SO_2_ bleaching or changes in wine pH, and are therefore desired for color stability (Boulton 2001). Similarly, interactions between the anthocyanin malvidin 3-monoglucoside and catechins in the presence of acetaldehyde, which also influence color and color stability in red wine, were observed (Rivas-Gonzalo, Bravo-Haro and Santos-Buelga 1995). The central role of acetaldehyde in these reactions even inspired researchers to experiment with exogenous addition of acetaldehyde, yielding red wines with reduced astringency and more stable color (Sheridan and Elias 2015).

Acetic acid is referred to, in industry, as volatile acidity or vinegar taint. While industrial *Saccharomyces* species can produce acetic acid, the presence of high acetic acid concentrations often indicates the presence of other species. High levels of acetic acid are typically associated with the respiratory metabolism of ethanol by acetic acid bacteria. However, some yeasts, notably *Brettanomyces* spp., can produce acetic acid in aerobic conditions (Crauwels *et al.*^2015^). This trait is highly strain and species dependent (Castro-Martinez *et al.*^2005^; Rozpędowska *et al.*^2011^). One species, *Brettanomyces bruxellensis*, is so efficient at producing acetic acid, it has been proposed as a candidate organism for industrial production (Freer 2002; Freer, Dien and Matsuda 2003).

In specific cases, the presence of these acid-producing species is desired for the fermentation, but more commonly acetic acid is a sign of spoilage. In wine, 0.2–0.4 g/L of acetic acid is acceptable, but above 1.2–1.3 g/L, it is considered a fault. In contrast, concentrations up to 1.5 g/L are common in Lambic beers and, in combination with bacterially produced lactic acid, are crucial for the sour characteristics of Lambic (Witrick 2012).

### Environmental parameter effects on acetaldehyde and acetic acid production

High levels of acetaldehyde are undesirable in an industrial context and some simple adjustments to fermentation parameters have been suggested to alter the level of acetaldehyde (Table 3). For example, acetaldehyde production in some wine strains remains constant when fermented between 12°C and 24°C but drastically increases at 30°C (Romano *et al.*^1994^). Supplementation of SO2 also induces acetaldehyde production, but the underlying mechanisms are unknown (Herraiz *et al.*^1989^; Herrero, García and Díaz 2003).

**Table 3.** Effect of environmental parameters on acetaldehyde, and acetic acid production.

**Parameter**	**Condition**	**Effect on acetaldehyde production**	**Reference**
**Temperature**	Increase	Increase	Romano *et al.* (1994)
**Oxygen**	Increase	Increase	Branyik *et al.* (2008), Curiel *et al.* (2016)
**Medium composition**			
***C source***	Non-fermentable	Increase	Romano *et al.* (1994)
***SO_2_***	Increase	Increase	Jackowetz *et al.* (2011)
		**Effect on acetic acid production**	
***Brettanomyces***			
**Oxygen**	Increase	Increase (direct effect on production)	Rozpedowska *et al.* (2011)
**Medium composition**			
***Antimicrobial agents (sulfite, chitosans, …)***	Supplementation	Decrease (inhibits growth)	Portugal *et al.* (2014)
***Weak acids and sorbic acid***	Supplementation	Decrease (inhibits growth)	Wedral *et al.* (2010)
**Low electric current**	Application of ∼200 mA	Decrease (inhibits growth)	Zuehlke *et al.* (2013)
**Pulsed electric field**	Application of ∼30 kV/cm, 1–4 μs pulses	Decrease (inhibits growth)	Zuehlke *et al.* (2013)
***Saccharomyces***			
**Temperature**	Decrease	Decrease	Beltran *et al.* (2008)
**Oxygen**	Increase	Increase	Curiel *et al.* (2016)
**Medium composition**			
***C concentration***	Increase	Increase (glycerol production, redox imbalance)	Bely *et al.* (2003)
***N source***	Supplementation	Decrease (stimulates yeast growth, provides NADH)	Bely *et al.* (2003), Barbosa *et al.*^2009^
***Copper***	Supplementation	Increase	Ferreira *et al.* (2006)
***Yeast lees and insoluble material***	Increase	Variable (some lead to increase, others to decrease)	Delfini and Costa (1993)

Since acetic acid has different sources in fermented beverages (yeast and bacteria), there are different strategies for targeting its production. Here we focus on control of yeast-derived acetic acid from two important yeast genera associated with industrial fermentations (Table 3). Production by *Brettanomyces* can be controlled by reducing oxygen availability (Rozpędowska *et al.*^2011^), supplementing the fermentation with antimicrobial agents (Portugal *et al.*^2014^) or applying electric currents (Zuehlke, Petrova and Edwards 2013). Production by *Saccharomyces* can be reduced by promoting general growth. Acetic acid production is driven by accumulation of NAD^+^ during glycerol production (Eglinton *et al.*^2002^) and increasing biomass (i.e. growth) can help regenerate the pool of NADH. Supplementation of nitrogen or unsaturated fatty acids can promote yeast growth with a subsequent reduction in acetic acid (Varela *et al.*^2012^). Reducing glycerol production by lowering the sugar concentration can also decrease the levels of acetic acid in the final product (Bely, Rinaldi and Dubourdieu 2003).

### Genetic factors and acetaldehyde and acetic acid production

Given the central role of acetaldehyde in carbon metabolism (Fig. 2), it is not a straightforward task to specifically modulate its production. However, attenuation of ethanol metabolism (Wang *et al.*^2013^), increasing acetaldehyde scavenging via glutathione (Chen *et al.*^2012^), oxidation of acetaldehyde to acetic acid (Yao *et al.*^2012^) or increasing pyruvate flux into the mitochondria (Agrimi *et al.*^2014^; Bender, Pena and Martinou 2015; Jayakody *et al.*^2016^) has been shown to reduce levels of acetaldehyde. Strains selected for resistance to Adh2 inhibitor 4-methylpyrazole demonstrated decreased *ADH2* expression and an 82% reduction in acetaldehyde production (Wang *et al.*^2013^). Similarly, direct disruption of *ADH2* reduces acetaldehyde by 68% (Wang *et al.*^2006^).

Reduction of volatile acidity is mainly a concern in the wine industry. Aerobic fermentation can cause excess levels of acetic acid. Due to the complexity of this part of the metabolic pathway, direct disruption of associated genes can have multiple and sometimes undesired effects. Deletion of *PDC1* or *ALD6* can reduce acetate levels but significantly increases levels of acetaldehyde, limiting its applicability (Luo *et al.*^2013^; Curiel *et al.*^2016^). The previously mentioned overexpression of *GPD1* effectively decreases ethanol production but also leads to excessively high acetic acid levels in wine (Cambon *et al.*^2006^). Combining this overexpression with deletion of *ALD6* reduces the acetic acid but also increases acetaldehyde and acetoin. This can be compensated by overexpression of *BDH1*, which diverts the excess acetaldehyde and acetoin to 2,3-butanediol, which has no effect on overall flavor and aroma (Fig. 2) (Ehsani *et al.*^2009^). Less direct approaches require less genetic compensation. For example, deletion of *AAF1*, a transcriptional regulator of the *ALD* genes, reduces acetic acid levels without affecting acetaldehyde production (Luo *et al.*^2013^). Strains with mutations in *YAP1*, a transcription factor involved in oxidative stress tolerance, also demonstrate reduced acetic acid levels (Yamamoto *et al*. 2000; Cordente *et al.*^2013^).

### Physiological and ecological roles of CO_2_, acetaldehyde and acetic acid

Though not a distinguishable aroma for humans, other organisms have distinct sensory responses to carbon dioxide. In yeast populations, including *S. cerevisiae*, CO_2_ can mediate cell–cell interactions, inducing growth and budding of neighboring colonies (Volodyaev, Krasilnikova and Ivanovsky 2013). In *Candida albicans*, increasing concentrations of self-generated CO_2_ causes the cells to undergo morphological changes and switch to hyphal growth (Hall *et al.*^2010^). Interestingly, this mechanism has been implicated in the pathogenicity of *C. albicans*, as the switch to filamentous growth is important for biofilm formation and invasive growth in the host (Hall *et al.*^2010^; Lu *et al.*^2013^).

Accumulation of acetaldehyde in yeast cells results in growth inhibition and a stress response (Stanley *et al.*^1993^; Aranda and Olmo 2004). When acetaldehyde diffuses out of the cell, it acts as a volatile signaling molecule. At high cell densities, yeast cells coordinate their metabolism by sensing the secreted acetaldehyde, resulting in collective macroscopic oscillations and synchronized phases of growth (Richard *et al.*^1996^). Interestingly, several cellular systems, from yeast colonies to human muscle, and even tumors, demonstrate this type of synchronized oscillations of glycolytic reactions (Betz and Chance 1965; Tornheim and Lowenstein 1974; Nilsson *et al.*^1996^; Richard 2003; Fru *et al.*^2015^).

Acetic acid is potentially used by *Brettanomyces* as a strategy to outcompete other microbes (Rozpędowska *et al.*^2011^). The ‘make-accumulate-consume’ strategy allows *Brettanomyces* yeast to accumulate high levels of acetic acid which dramatically lowers the pH of the environment. Since this yeast has a higher tolerance for low pH than most microbes, it can withstand the extreme environment and later consume the acetic acid as an extra carbon source.

These three compounds also play an important role in insect behavior. Acetaldehyde is a core component of a compound blend used to attract and trap pest beetles from the genus *Carpophilus* (Phelan and Lin 1991; Nout and Bartelt 1998). In several reports, CO_2_ had a repulsive effect on fruit flies (Suh *et al.*^2004^; Turner and Ray 2009). Recent studies indicate that this repulsion highly depends on the behavioral context, i.e. whether the flies are walking on surface or flying in the air (Wasserman, Salomon and Frye 2013). When in flight, *Drosophila melanogaster* are attracted to CO_2_, possibly due to modulations of neurotransmitters which occur during flight (Orchard, Ramirez and Lange 1993). The current hypothesis is that in crowded conditions, when flies are gathered on a surface, CO_2_ is repulsive but when in flight and searching for food, CO_2_ can act as an attractive signal to indicate the presence of fermenting fruits.

Acetic acid is also an important volatile for mediating the behavior of *D. melanogaster*. This fruit fly is reported to have a highly selective olfactory neuron for detection of acids which is generally connected with observed acid-avoiding behavior (Ai *et al.*^2010^). However, *D. melanogaster* is also known to be lured by acetic acid (Hutner, Kaplan and Enzmann 1937; Knaden *et al.*^2012^), which accounts for its attraction to vinegar and nickname as the ‘vinegar fly’. Females looking for ovipositioning sites are strongly attracted by acetic acid, whereas flies not ready to deposit eggs show little or no attraction (Joseph *et al.*^2009^; Gou *et al.*^2014^). The closely related species, *D. simulans*, is repulsed by microbially produced acetic acid; this behavior strongly correlates with the increasing acid concentration (Günther *et al.*^2015^). These examples suggest a complexity in the perception and processing of sensory information, both gustatory and olfactory, to modulate behavior. In this example, it has been hypothesized that the egg-laying preference on acetic acid-containing substrates depends on gustatory inputs (females will taste the acetic acid when on the surface). However, when not in direct contact with the medium, olfactory information only leads to aversion of acetic acid-containing food (Joseph *et al.*^2009^). Together with ethanol, acetic acid has also been found as an important volatile to attract flies such as *Fannia canicularis, Muscina stabulans* and *Philornis downsi* (Diptera: Muscidae) to fermenting substrates as a food source (Landolt, Cha and Zack 2015; Cha *et al.*^2016^). Furthermore, when acetic acid is combined with other fermentation compounds, such as phenylacetaldehyde, stronger attraction of insects is achieved (Becher *et al*. 2010, 2012; Cha *et al.*^2012^).

## AMINO ACID METABOLITES: VICINAL DIKETONES

### Biochemistry of vicinal diketone production

**Figure 3.**
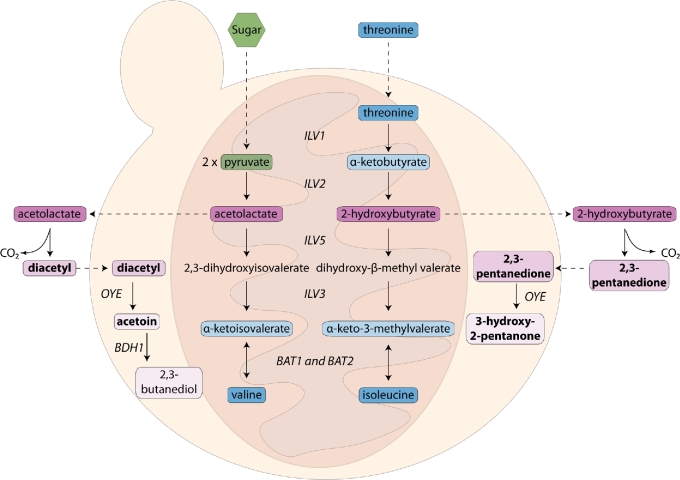
**Production of vicinal diketones**. The vicinal diketones are produced as by-products during the isoleucine-leucine-valine (ILV) biosynthetic pathways. Gene names correlate with nomenclature from *S. cerevisiae* (Saccharomyces Genome Database). OYE = ‘Old Yellow Enzyme’. Dotted lines indicate import/export, solid lines indicate biochemical reactions. Note: dotted line from sugar to pyruvate also encompasses glycolysis.

Vicinal diketones (i.e. compounds containing two adjacent carbon-oxygen double bonds) can be produced during fermentation through non-enzymatic decarboxylation of intermediates in the valine and isoleucine anabolic pathways (Fig. 3). During fermentation, pyruvate can be converted to various carbon compounds such as acetolactate. The acetolactate can then be diverted towards synthesis of valine and leucine. Inefficiency of the valine biosynthesis pathway during growth results in a buildup of acetolactate which is then secreted into the medium. Similarly, during isoleucine biosynthesis, acetohydroxybutyrate is produced and is also secreted. Both compounds are non-enzymatically converted to diketones: decarboxylation of acetolactate forms diacetyl (2,3-butanedione) while decarboxylation of acetohydroxybutyrate forms 2,3-pentanedione. Towards the end of fermentation, these compounds can be reabsorbed by the cell and converted to acetoin (and subsequently 2,3-butanediol) and 3-hydroxy-2-pentanone by various reductases (van Bergen *et al.*^2016^).

### Vicinal diketones in industry

Vicinal diketones can provide a pleasant nutty, toasty and toffee-like flavor in fermented foods and beverages, most notably beer, wine and dairy products (Molimard and Spinnler 1996; Bartowsky and Henschke 2004; Krogerus and Gibson 2013a). However, they are considered off-flavors when present in high concentrations, changing their sensory perception to ‘buttery’ or ‘rancid’. Especially in beer brewing, vicinal diketone production is an ongoing challenge. Diacetyl is rarely perceived positively in beer, except in a few specific styles (e.g. sour ales, Bohemian Pilsner and some English ales).

Diacetyl is generally more of a focus in industrial beer fermentation than 2,3-pentanedione for two reasons. First, it has a significantly lower sensory threshold (0.1 μg/g versus 1.0 μg/g) which makes it more detectable in the final product. Second, the direct connection between diacetyl and pyruvate has implications in managing ethanol production levels. In wine, diacetyl is considered less of a problem and low (1–4 μg/g) concentrations positively contribute to desirable buttery or butterscotch notes. Moreover, excessively high concentrations are rare but rather indicate bacterial spoilage or other irregularities during malolactic fermentation (Bartowsky and Henschke 2004). Additionally, diacetyl is masked in part by the presence of SO_2_ in wine which results in a marked increase in threshold levels (Bartowsky and Henschke 2004).

### Environmental parameters and vicinal diketone production

Due to the highly reductive conditions that exist at the end of alcoholic fermentations, the concentration of diacetyl is usually below (or close to) its sensory detection threshold in fresh beer (Haukeli and Lie 1972). Diacetyl reduction effectively eliminates the undesired flavors as acetoin and 2,3-butanediol do not contribute to the aroma profile. Therefore, some beers are subjected to a maturation phase of 2–3 weeks after fermentation to allow any residual acetolactate to decarboxylate and subsequently be reduced by the yeast to below its detection limit. This maturation phase requires storage capacities and limits the output of beer from a brewery and the economic feasibility. Therefore, there have been some considerable efforts to find alternative ways to reduce natural diacetyl formation or speed up diacetyl reduction by modifying various process parameters (Table 4).

**Table 4.** Effect of environmental parameters on vicinal diketone production.

**Parameter**	**Condition**	**Effect on vicinal diketone production**	**Reference**
**Temperature**	Increase	Decrease during fermentation or maturation (higher cell density, more acetolactate to diacetyl conversion)	Bamforth and Kanauchi (2004)
**pH**	Decrease	Increase (increased enzyme efficiency)	Bamforth and Kanauchi (2004)
**Fermentation time**	Increase	Decrease (more acetolactate to diacetyl conversion and diacetyl reduction)	Bamforth and Kanauchi (2004)
**Oxygen**	Increase	Decrease (higher cell density)	Portno (1966)
**Medium composition**			
***Valine supplementation***	Increase	Decrease (less acetolactate production, see Figure 2)	Krogerus and Gibson (2013b)
***Sugar concentration***	Increase	Decrease	Saerens *et al.* (2008b)
**Enzyme (α-Acetolactate decarboxylase)**	Supplementation	Decrease (acetolactate to acetoin conversion)	Godtfredsen and Ottesen (1982)

The connection to amino acid metabolism directly affects synthesis of these two compounds; if nitrogen is low and the cell needs to synthesize its amino acids, production of these by-products will also increase (Krogerus and Gibson 2013a). Simply supplementing fermentation media with exogenous valine can dramatically decrease production of diacetyl (Krogerus and Gibson 2013b). Since the conversion of acetolactate to diacetyl is non-enzymatic, heating after fermentation increases the rate of conversion of excess acetolactate, which can subsequently be reduced (Kobayashi *et al.*^2005^). The use of a continuous fermentation setup minimizes yeast growth, and thus valine biosynthesis, and reduces formation of diacetyl (Verbelen *et al.*^2006^).

### Genetic factors and vicinal diketone production

Arguably one of the most promising and cheaper strategies to reduce vicinal diketones is modification of yeast metabolism. Most commonly, this is done by increasing the metabolic flux from acetolactate to valine or promoting conversion of acetolactate to acetoin. Mutation of *ILV2* (acetolactate synthase) reduces diacetyl formation by 64% (Wang *et al.*^2008^). Similarly, increased expression of *ILV5* (acetohydroxyacid reductosiomerase), the rate-limiting step in valine synthesis, reduces diacetyl formation 50%–60% (Mithieux and Weiss 1995; Kusunoki and Ogata 2012). Heterologous expression of a bacterial acetolactate decarboxylase gene (ALDC) catalyzes the non-oxidative decarboxylation of acetolactate to acetoin and bypasses diacetyl production (Kronlof and Linko 1992).

### Physiological and ecological roles of vicinal diketones

As described, production of the vicinal diketones is done extracellularly following the secretion of accumulated acetolactate and acetohydroxybutrate. The biological role of this phenomenon is not known, but protection from carbonyl stress and subsequent cellular damage has been suggested (van Bergen *et al.*^2016^). Additionally, the reduction of the diketones is physiologically favorable for yeast, as the resulting end products are less toxic and the reactions replenish the NAD^+^ and NADP^+^ pools (De Revel and Bertrand 1994).

Diacetyl has a ‘masking’ role in ecological settings rather than a direct role as a signaling molecule. *Drosophila melanogaster* has high specificity neurons for detecting diacetyl and CO_2_ (de Bruyne, Foster and Carlson 2001). As discussed earlier, CO_2_ can elicit avoidance behavior in fruit flies, which seems somewhat counterintuitive, since CO_2_ is a signal of fermenting fruit, a suitable food source and ovipositioning site. Diacetyl masks the avoidance signal by blocking the receptor, resulting in attraction to the fermentation source (Turner and Ray 2009; Turner *et al.*^2011^). A reversed interplay is observed in several mosquito species, where mosquitoes are attracted to CO_2_ which is then blocked by the presence of diacetyl (Turner *et al.*^2011^).

## AMINO ACID METABOLITES: HIGHER ALCOHOLS

**Figure 4.**
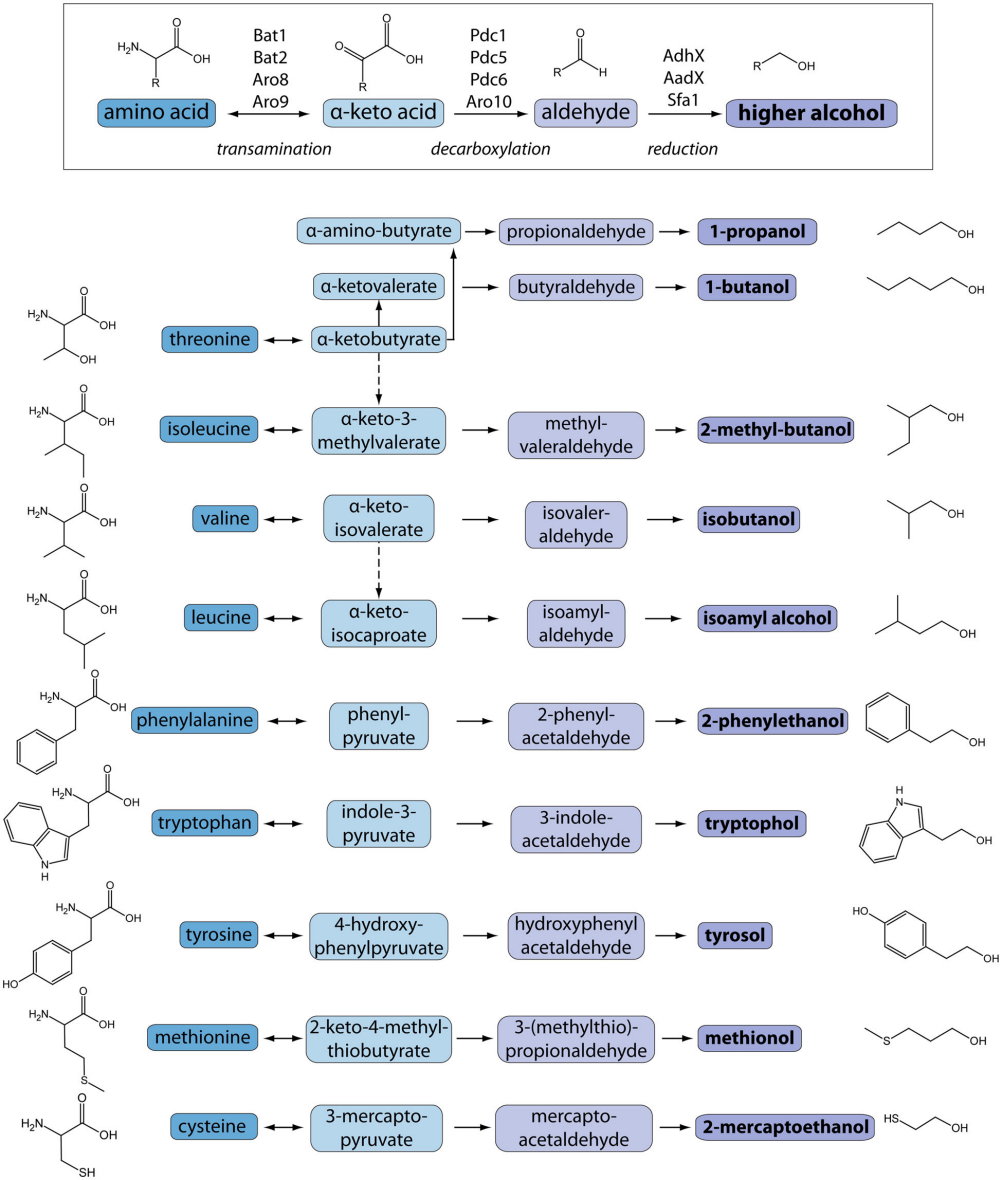
**The Ehrlich pathway**. There are several routes that can direct carbon compounds into the production of amino acids and subsequently the higher alcohols. This scheme depicts the most direct connections between the amino acids and the respective higher alcohols through the three-step Ehrlich Pathway (general reactions depicted at top). Dotted lines indicate multiple steps. Note: the reduction step can be carried out by over 10 different enzymes which vary in localization, regulation and substrate specificity; AdhX = alcohol dehydrogenase (Adh1, Adh2, Adh3, Adh4, Adh6, Adh7); AadX = aryl alcohol dehydrogenase (Aad3, Aad4, Aad6, Aad10, Aad14, Aad15, Aad16).

Perhaps the most well-characterized biochemical pathway in yeast aroma production is the Ehrlich pathway. This is likely due to the very desirable and recognizable compounds produced by this pathway—the higher (fusel) alcohols and subsequently, the acetate esters. Felix Ehrlich first posited the connection between amino acid metabolism and higher alcohol formation in 1907 based on their structural similarity (Fig. 4). This led to a simple, classic experiment of varying the concentration of specific amino acids in the fermentation media and noting changes in production of the corresponding fusel alcohols (Ehrlich 1907). Over the next century, the details of this biochemical process have been greatly uncovered leading to significant improvements in the fermentation industry.

### Biochemistry of higher alcohol production

The Ehrlich pathway is a three-step process that modifies assimilated amino acids, the major source of nitrogen in many fermentation processes. In general, amino acids are deaminated, decarboxylated and finally reduced to their respective alcohol derivatives (Fig. 4). By sequentially modifying amino acids, yeast cells can harvest and utilize the essential nitrogen as needed and in turn produce an array of fragrant and distinct aroma compounds (Hazelwood *et al.*^2008^; Pires *et al.*^2014^). Given the chemical similarities of the intermediates to pyruvate, acetaldehyde, and ethanol, many of the same enzymes involved in production of the primary fermentation metabolites are also involved in this pathway.

#### Transamination

After uptake from the media, amino acids are converted to their respective α-keto acid by a transaminase capable of transferring amine groups between amino acids. In *Saccharomyces cerevisiae*, there are six enzymes capable of this type of reaction: Bat1, Bat2, Aat1, Aat2, Aro8 and Aro9 (SGD, Cherry *et al.*^2012^). Aat1 and Aat2 do not play a role in higher alcohol production; these enzymes act specifically on aspartate as part of the malate-aspartate shuttle to move electrons from the cytosol to the mitochondria for respiratory energy production (Cronin *et al.*^1991^; Morin, Subramanian and Gilmore 1992). The other four enzymes have been directly linked to higher alcohol synthesis but, as seen with the ADHs discussed above, each contributes differently to the Ehrlich pathway. Bat1 and Bat2 are primarily involved with transamination of the branched chain amino acids, whereas Aro8 and Aro9 are aromatic amino acid transaminases acting on phenylalanine and tryptophan, respectively (Kispal *et al.*^1996^; Iraqui *et al.*^1998^).

#### Decarboxylation

The second step of the Ehrlich pathway is the irreversible decarboxylation of the α-keto acid to an aldehyde. The same three PDCs used in the production of acetaldehyde (Pdc1, Pdc5 and Pdc6) have all been implicated in the production of the fusel aldehydes. Additionally, Aro10 is capable of this reaction, and is primarily responsible for decarboxylating 2-phenylpyruvate to 2-phenylacetaldehyde (Vuralhan *et al.*^2003^). Aro10 is also a likely candidate for some variations in higher alcohol production between species. *Saccharomyces kudriavzevii* produces more higher alcohols than *S. uvarum* or *S. cerevisiae* (Stribny *et al.*^2016^). *Sc*Aro10 prefers phenylpyruvate but *Sk*Aro10 has a broader substrate preference, almost equally acting on phenylpyruvate, ketoisocoaproate, ketoisovalerate, ketomethylvalerate and even keto-γ-methylthiobutyrate (Stribny *et al.*^2016^). Interestingly, the interspecies hybrid, *S. pastorianus*, harbors three copies of the *S. cerevisiae ARO10* gene and one copy from *S. eubayanus*. While both isozymes prefer phenylpyruvate as a substrate, *Seu*Aro10 has much higher activity towards ketoisovalerate (Bolat *et al.*^2013^). Copy number variation and slight discrepancies in substrate preference add a level of aroma complexity to hybrid brewing yeasts.

#### Reduction

At this point, the fusel aldehydes can undergo an oxidation or a reduction. The various ADHs and AADs catalyze the reduction step and complete the Ehrlich pathway. Any one of the ADH enzymes can catalyze this last step, but research indicates that Adh1 and Adh2 mainly participate in ethanol metabolism (described above). If the fusel aldehydes undergo an oxidation reaction by an ALD, they are converted into their respective fusel acids.

### Higher alcohols in industry

Higher alcohols can impart a much-desired effect on the product's flavor despite their higher sensory threshold, which can differ several orders of magnitude compared to their corresponding acetate esters. The major fusel alcohols found in alcoholic beverages are 1-propanol (alcoholic aroma), 1-butanol (alcoholic), isobutanol (alcoholic), 2-phenylethanol (roses, flowery) and isoamyl alcohol (banana, fruity).

The rose-like fragrance of 2-phenylethanol has made it a desirable compound for use in many perfumes, cosmetics and beverages (Etschmann *et al.*^2002^). Currently, the greater part of its commercial production is done synthetically, but this process requires use of carcinogenic precursors, such as benzene and styrene, and yields various difficult-to-remove by-products. It is possible to extract 2-phenylethanol from the essential oils of plants, but this process is excessively expensive due to low yields (Etschmann *et al.*^2002^). Therefore, researchers have turned to microbial production of this compound (Carlquist *et al.*^2015^). Genetically modified or mutagenized *Saccharomyces cerevisiae* strains have been utilized to convert phenylalanine into 2-phenylethanol, typically by enhancing the Ehrlich pathway (Kim, Cho and Hahn 2014). Non-conventional yeasts have also been explored as production strains including *Kluyveromyces marxianus*, which naturally produces more 2-phenylethanol than *S. cerevisiae* (Ivanov *et al.*^2013^). Additionally, *K. marxianus* grows quickly and is thermotolerant making it an interesting candidate for commercial production (Etschmann, Sell and Schrader 2003; Gao and Daugulis 2009; Morrissey *et al.*^2015^).

The associated fusel acids are also of industrial interest. The production of these compounds can be perceived positively or negatively depending on the context. In soy sauce, flor-forming strains of *Zygosacharomyces rouxii* can produce 2-methylpropanoic acid (isobutyric acid) and 3-methylbutanoic acid (isobutyric acid) (corresponding alcohols isobutanol and isoamyl alcohol), compounds associated with foul, spoiled aromas. In some cases, metabolic engineering approaches have been employed to actually increase production of these acids. Short branched-chain fatty acids such as 2-methylbutanoic acid, isobutyric acid and isovaleric acid are valuable compounds in the food and pharmaceutical industries. The acids and their derivatives can be used as fragrances and flavorings (Yu *et al.*^2016^).

### Environmental parameters and higher alcohol production

The three-step process described above is situated amongst a complex network of amino acid metabolism: there are multiple paths to each of the major alcohols that require significant regulation and balance during the fermentation process. Additionally, levels of each compound are dramatically affected by the medium composition, especially carbon source and nitrogen sources (Table 5). Since higher alcohols are mainly produced during active growth, factors that positively influence yeast growth simultaneously promote higher alcohol synthesis (Dekoninck 2012). If there is a surplus of exogenous amino acids, as shown by Ehrlich and others, production of these alcohols increases (Ehrlich 1907; He *et al.*^2014^). If amino acids are in short supply, the pathways will inevitably favor anabolic routes. This understanding has been adopted by industry as a powerful way to direct higher alcohol production (Etschmann *et al.*^2002^; Vidal *et al.*^2013^; Lei *et al.*2013a).

**Table 5.** Effect of environmental parameters on higher alcohol production.

**Parameter**	**Condition**	**Effect on higher alcohol production**	**Reference**
**Temperature**	Increase	Increase	Landaud *et al.* (2001)
**Oxygen**	Increase	Increase	Valero *et al.* (2002)
**Medium composition**			
***C source***	Maltose	Decrease (compared to sucrose, fructose, glucose)	Younis and Stewart (1998)
***Sugar concentration***	Increase	Decrease (not always)	Younis and Stewart (1999)
***N source (total)***	Increase	Decrease (co-regulation of *LEU* and *BAT* genes)	Yoshimoto *et al.* (2002)
***NH_4_***	Supplementation	Decrease	Vidal *et al.* (2013)
***Amino acids***	Supplementation	Increase in respective higher alcohol (see Fig. 3)	Hernandez-Orte *et al.* (2005)
***Vitamins***	Supplementation	Increase	Etschmann *et al.* (2004)
***Maillard compounds***	Increase	Increase	Dack *et al.* (2017)

### Genetic factors and higher alcohol production

Engineered yeast strains for increased higher alcohol production are utilized both for increasing concentrations of the alcohols themselves and their respective esters. Overexpression of *ADH6* can increase isobutanol production (Kondo *et al.*^2012^) whereas overexpression of *ADH1* can increase levels of 2-phenylethanol (Shen *et al.*^2016^). Our understanding of the ILV biosynthetic and Ehrlich pathways allows for complex, multistep metabolic engineering to increase specific higher alcohols. For example, overexpression of *ILV2, ILV3, and ILV5* increases the flux towards isoleucine production (Fig. 3). If this is coupled with deletion of *BAT1* (transaminase) and *ALD6* (the aldehyde dehydrogenase) plus overexpression of *ARO10* and *ADH2* (both alcohol dehydrogenases)**, the α-keto acid and aldehyde derivatives of isoleucine are pushed towards production of the higher alcohol (Fig. 4) (Park, Kim and Hahn 2014). Conversely, deletion of the alcohol dehydrogenase *ADH* with overexpression of *BAT1, ALD2* and *ALD5* increases the production of the fusel acids by diverting flux at the last Ehrlich step towards oxidation. These acids are also intermediates for production of value-added products in the chemical industry (Yu *et al.*^2016^).

Due to the complexity and intricate nature of these pathways, simple mutation does not always have the desired effect. For example, some studies show that deletion of *ARO8* (one of the aromatic amino transferases) increases catabolism of phenylalanine to its higher alcohol 2-phenylethanol (Romagnoli *et al.*^2015^; Shen *et al.*^2016^) while others have demonstrated that overexpression of the same gene also increases production of higher alcohols (Yin *et al.*^2015^; Wang *et al.*2016b). Additionally, deletion of *ARO9* has no apparent effect (Shen *et al.*^2016^) but its overexpression causes an increase in production of higher alcohols (Kim, Cho and Hahn 2014). These conflicting results could be due to a multitude of factors including differences in strain background or variations in media used for fermentations. Regardless, this points to a significantly more complicated relationship between the aminotransferases that may help contribute to the diversity of higher alcohol production in different strains.

As becomes apparent from the previous examples, sophisticated metabolic engineering is needed to obtain highly productive strains for higher alcohols. Several research teams focus on butanol isomers as these compounds can be used as alternative fuels. An exhaustive overview of metabolic engineering strategies for butanol isomer production has recently been published elsewhere (Generoso *et al.*^2015^). But, despite the extensive efforts to improve the production yield of butanol isomers (and higher alcohols in general) in *S. cerevisiae*, the efficiency that can be achieved by metabolic engineering is still significantly lower compared to other hosts, such as *Escherichia coli*. Comparison of central metabolism of metabolically engineered *E. coli* and *S. cerevisiae* revealed that flexibility of this metabolism is an important factor in efficient production of butanols and propanols (Matsuda *et al.*^2011^).

### Physiological and ecological roles of higher alcohols

Given the significant variation in higher alcohol production from different yeasts, it is perhaps not surprising that insects have developed an ability to utilize these compounds as chemical signatures. Many insect olfactory receptors are specifically attuned to the detection of higher alcohols and many of these compounds can elicit antennal and behavioral responses in insects (Hallem and Carlson 2004; Saerens, Duong and Nevoigt 2010; Knaden *et al.*^2012^; Witzgall *et al.*^2012^). It has been demonstrated on several occasions that cultures of the yeast-like fungus *Aureobasidium pullulans* can lure a variety of insects, including hoverflies (Diptera: Syrphidae) (Davis and Landolt 2013) and social wasps (*Vespula* spp. (Hymenoptera: Vespidae) (Davis, Boundy-Mills and Landolt 2012). In both cases, a synthetic blend of higher alcohols, namely 2-methyl-1-butanol, isoamyl alcohol and 2-phenylethanol, proved to be even more attractive to the insects than the yeast culture. The wasps are known to act as vectors for *A. pullulans*, suggesting a strong interaction between the wasps and the yeast species (Davis, Boundy-Mills and Landolt 2012).

Compound blends to mimic fermenting yeasts are commonly being implemented to combat agricultural pests. Many of the blends contain higher alcohols since these tend to assist in eliciting antennal responses and attraction. The beetle *Carpophilus humeralis* infests and damages corn crops, and higher alcohol-containing blends are designed to mimic *S. cerevisiae* fermenting corn and lure them (Nout and Bartelt 1998). The related beetle, *C. hemipterus*, is similarly attracted to *S. cerevisiae*-produced higher alcohols, namely 2-pentanol, isoamyl alcohol, isobutanol and butanol (Phelan and Lin 1991). The weevil *Araecerus fasciculatus* (Coleoptera: Anthribide), a coffee bean pest, was recently found to be attracted to 2-phentylethanol implying that the compound might serve as a potential lure (Yang *et al.*^2016^).

Higher alcohols can also serve as directory signals for insects. Fermentations of *S. cerevisiae* or a synthetic blend of five fermentation compounds, including ethanol, isoamyl alcohol and 2-phenylethanol, is sufficient to attract *D. melanogaster* (Becher *et al.*^2012^). Among other compounds, higher alcohols produced by *Metschnikowia*, including isoprenol, 2-phenylethanol and citronellol, can elicit antennal responses in the codling moth *Cydia pomonella* (Lepidoptera: Tortricidae) (Witzgall *et al.*^2012^). The moths utilize the emitted aromas to orient themselves towards suitable oviposition sites, such as yeast-infested apples that provide a food source for larvae and protection from harmful fungal infestations.

Some higher alcohols have antifungal properties. Isoamyl alcohol produced by *Candida maltosa* inhibits the germination of filamentous fungi (Ando *et al.*^2012^). *Pichia anomala* produces 2-phenylethanol potentially as a biocontrol agent against *Aspergillus flavus;* the compound inhibits spore germination and the production of the carcinogenic mycotoxin produced which can contaminate the crops *P. anomala* grows on (Hua *et al.*^2014^). *Kloeckera apiculata* likewise produces 2-phenylethanol to inhibit growth of various *Penicillium* molds (Liu *et al.*^2014^). Other studies have also demonstrated anti-fungal effects of yeast volatiles from various species (several *Candida* species*, S. cerevisiae*, *A. pullulans, Metschnikowia pulcherrima*), but the specific effector compounds have not yet been identified (Fiori *et al.*^2014^; Parafati *et al.*^2015^; Lemos Junior *et al.*^2016^).

Several higher alcohols such as 2-phenylethanol, tryptophol, tyrosol and farnesol can act as quorum-sensing molecules in dimorphic yeasts, including *S. cerevisiae*, *Debaryomyces hansenii* and *Candida albicans.* Secretion of the alcohols regulates the switch between unicellular yeast forms and filamentous forms (Chen *et al.*^2004^; Chen and Fink 2006; Gori *et al.*^2011^). Moreover, it has been speculated that these quorum-sensing molecules can play a role on the population level and influence the establishment of microbial communities in (semi-) spontaneous fermentations, such as wine, lambic beers and/or cheese, but evidence for such interactions is still lacking (Ciani and Comitini 2015).

## AMINO ACID METABOLITES: ESTERS

### Biochemistry of ester production

**Figure 5.**
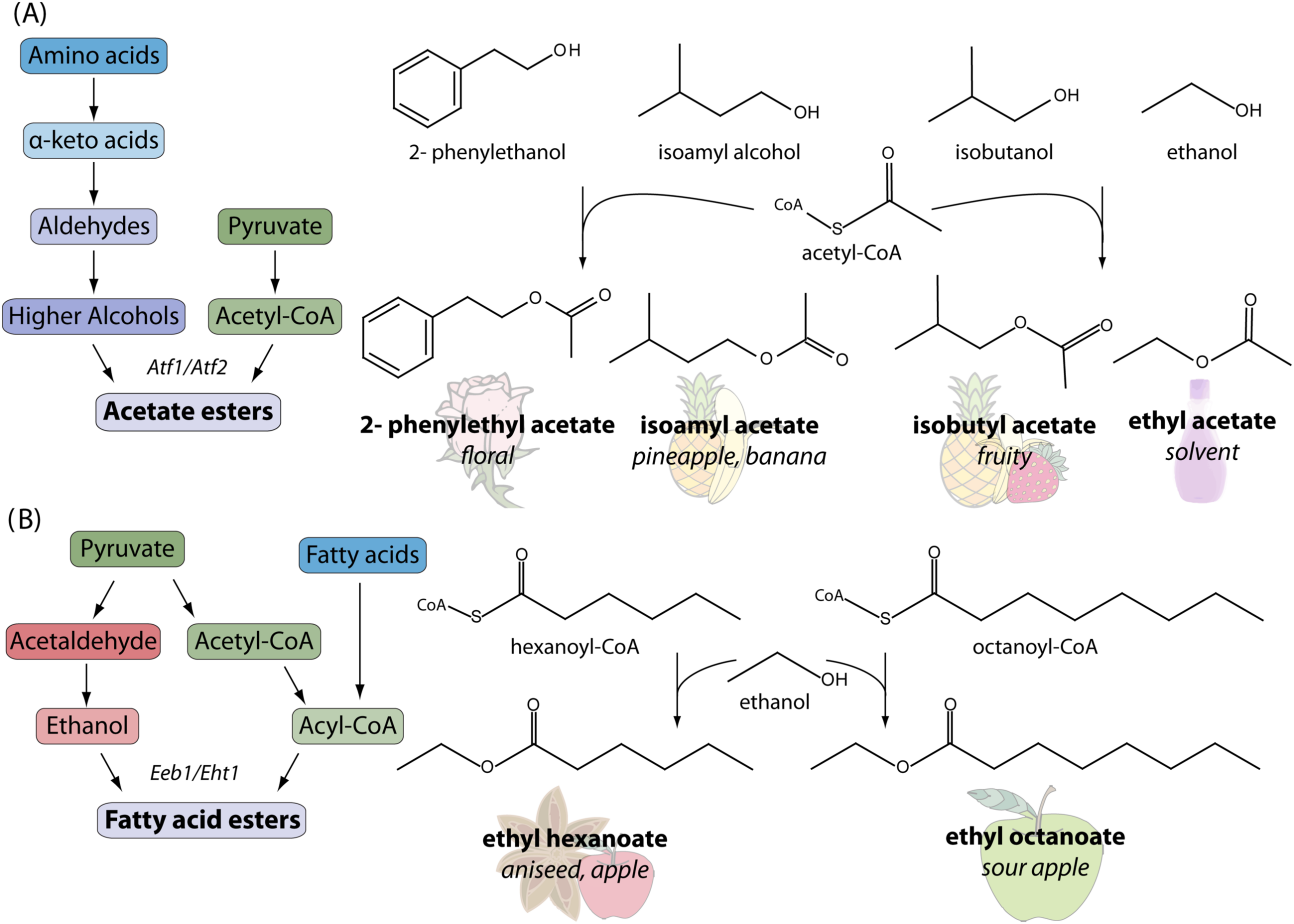
**Ester synthesis in yeast. Left: general scheme of both types of ester production**. Esters are the result of condensation reactions between an alcohol and an acetyl/acyl-CoA. (**A**) Acetate esters are produced through the actions of Atf1 and Atf2. (**B**) Fatty acid esters are produced by Eeb1 and Eht1. Right: examples of some of the most common esters discussed in this review. General aroma descriptors are listed in italics.

Esters are formed by a condensation reaction between acetyl/acyl-CoA and an alcohol (Fig. 5). The use of acetyl-CoA or acyl-CoA divides esters into two different categories, acetate esters and fatty acid ethyl esters, respectively. The small size and lipophilic nature of acetate esters allow them to readily diffuse from the cytoplasm into the extracellular medium whereas the longer hydrocarbon tails of fatty acid ethyl esters reduce their capacity to diffuse across the membrane. Therefore, acetate esters impart significantly more influence over flavor and fragrance than the fatty acid counterparts.

Ester synthesis is carried out by alcohol-O-acetyl (or acyl)-transferases (AATases). In *Saccharomyces cerevisiae*, there are four known enzymes: Atf1 and Atf2 are responsible for most acetate ester production and Eeb1 and Eht1 synthesize the fatty acid ethyl esters (SGD, Cherry *et al.*^2012^). There is definitive evidence that there are additional enzymes of both types in *S. cerevisiae*. Double deletion of *ATF1* and *ATF2* results in complete loss of isoamyl acetate production but only a 50% reduction in ethyl acetate (Verstrepen *et al.*2003c). Similarly, a double deletion of *EEB1* and *EHT1* does not eliminate fatty acid ethyl esters (Saerens *et al.*^2006^).

Recently, a third ethyl acetate-forming enzyme has been described (Kruis *et al.*^2017^). The ethanol acetyltransferase 1 (Eat1) was identified in *Wickerhamomyces anomalus* and defines a new family of enzymes which is distinct from the canonical AATases. Eat1 is actually a hydrolase that can perform thioesterase and esterase reactions in addition to formation of ethyl acetate. Homologs are found in several ethyl acetate-producing yeasts. Although a triple deletion has not yet been attempted, deletion of the *S. cerevisiae* Eat1 homolog, *YGR015C*, results in a 50% reduction in ethyl acetate production, which complements the Atf1 and Atf2 production.

The enzymatic activities of these enzymes can differ significantly, even more so between different species and strains, adding to the variation of the final fermentation product. For example, Atf1 has equal substrate specificity for isoamyl alcohol and 2-phenylethanol whereas Atf2 prefers isoamyl alcohol (Stribny *et al.*^2016^). However, both Atf1 and Atf2 from *S. kudriavzevii* or *S. uvarum*, have higher preference for 2-phenylethanol compared to the *S. cerevisiae* homologs. This is directly reflected under fermentation conditions, where strains harboring *S. kudriavzevii* and *S. uvarum* enzymes produce much more 2-phenylethyl acetate.

### Esters in industry

Esters are generally accepted as some of the most important contributors to the flavor and aroma of alcoholic beverages, imparting fruity and flowery notes to the product (Nordström 1966; Verstrepen *et al.*2003a). During industrial fermentations, yeasts produce esters in very low concentrations, often only a few parts per billion (ppb) (Lambrechts and Pretorius 2000). Incidentally, these natural concentrations hover around the flavor threshold for humans and consequently, small changes in ester production can significantly alter perception of the product. There is a synergistic effect in the perception of many esters, where a mixture of compounds will highlight or mask the presence of others (Nordström 1964a; Suomalainen 1971). However, an excess of esters often results in an unpalatable product, highlighting the importance of balance in the production of aroma compounds (Liu, Holland and Crow 2004).

The overall importance and complexity of ester production has led to considerable industrial research to optimize production. Interestingly, these compounds affect the quality of practically all food fermentations that involve yeasts, ranging from fermented beverages (Lilly, Lambrechts and Pretorius 2000; Verstrepen *et al.*2003a), over bread (Birch *et al.*^2013^; Aslankoohi *et al.*^2016^), to chocolate (Meersman *et al.*^2016^). Moreover, biotechnological production of high ester concentrations, especially ethyl acetate, has been studied for several years. Ethyl acetate is an environmentally friendly solvent with many industrial applications but its production involves energy-intensive petrochemical processes. Several non-conventional yeasts, more specifically *W. anomala*, *Candida utilis* and especially *Kluyveromyces marxianus*, all species with inherently high ethyl acetate production, have been explored (Löser, Urit and Bley 2014).

### Environmental parameters and ester production

There are a multitude of parameters that can influence ester production in yeast which allows for significant modulation of the ester profile of foods or beverages without genetic manipulation (Table 6). However, given the complexity of the regulation of enzyme and substrate availability, the exact outcome of modifying one specific parameter is still hard to predict. In general, acetate and ethyl ester production are often affected in the same way by the same parameters (Saerens *et al.*2008a).

**Table 6.** Effect of environmental parameters on ester production.

**Parameter**	**Condition**	**Effect on ester production**	**Reference**
**Temperature**	Increase	Increase (not always)	Molina *et al.* (2007), Saerens *et al.* (2008a)
**Oxygen**	Increase	Decrease (decreased expression of ester synthesis genes)	Fujii *et al.* (1997), Anderson and Kirsop (1974)
**Medium composition**			
***Unsaturated fatty acids***	Increase	Decrease (decreased expression of ester synthesis genes)	Fujii *et al.*^1997^, Anderson and Kirsop (1974)
***Free amino nitrogen (FAN)***	Increase	Increase (precursor availability and increased expression of ester synthesis genes)	Saerens *et al.* (2008a), Lei *et al.* (2013ba)
***Sugar concentration***	Increase	Increase (increased expression of ester synthesis genes)	Saerens *et al.* (2008b)
***C source***	Glucose, fructose, sucrose	Increase (compared to maltose)	Verstrepen *et al.* (2003b), Piddocke *et al.* (2009)
***Maillard compounds***	Increase	Decrease	Dack *et al.* (2017)
**Hydrostatic pressure**	Increase	Decrease (increased dissoved CO_2_)	Landaud *et al.* (2001), Meilgaard (2001)

The concentration and composition of fermentable carbon sources as well as the carbon-to-nitrogen ratio have dramatic effects on ester production (Table 6) (Piddocke *et al.*^2009^; Dekoninck *et al.*^2012^). The direct connection to higher alcohols and their amino acid precursors makes ester production highly dependent on the nitrogen source. The concentration of free amino nitrogen (FAN), including amino acids and small peptides, positively correlates with acetate ester production (Procopio *et al.*^2013^; Lei *et al.*2013a, b). Increased nitrogen can also increase expression of *ATF1* and *BAT1* and subsequently alter ester levels (Saerens, Thevelein and Delvaux 2008).

In general, higher temperatures result in higher alcohol production and subsequent acetate ester production (Landaud, Latrille and Corrieu 2001) though this effect can vary given differences in fermentation matrix, genetic background and the esters of interest (Molina *et al.*^2007^; Birch *et al.*^2013^). Additionally, *ATF1* and *ATF2* expression are positively correlated with temperature and would result in increases in acetate ester production (Saerens *et al.*2008b). However, the volatile nature of acetate esters would lead to an overall decrease in concentration at excessively high temperatures. This is the case in chocolate production; during post-fermentation processing, the chocolate mass is subjected to an hour-long mixing at temperatures as high as 75°C (Meersman *et al.*^2016^). This production step results in the loss of many yeast-derived aroma compounds, including acetate esters. However, fatty acid esters, which dissolve more easily into the fat fraction of chocolate, are largely retained.

Dissolved oxygen and unsaturated fatty acids are negative regulators of *ATF1* expression and, consequently, ester synthesis (Dufour, Malcorps and Silcock 2003). Interestingly, both compounds are shown to act on the same part of the *ATF1* promotor, namely the low-oxygen response element (Jiang *et al.*^2001^). Therefore, oxygenation of the fermenting medium is a powerful and straightforward tool to modulate ester production. However, it is not always feasible to increase or decrease the oxygen content of the medium, as it can have undesirable side effects (e.g. irregular yeast growth, impaired flavor stability or increased risk of contamination). Adding unsaturated fatty acids can be an interesting alternative without the undesired effects (Moonjai *et al.*^2002^).

Modifications to the fermentation vessel can alter the yeast cells’ microenvironment and affect physiological changes. A shift from small fermenters to tall, cylindroconical vessels in large breweries resulted large decreases in ester production (Meilgaard 2001). This was explained by the increased concentration of dissolved carbon dioxide which inhibited overall decarboxylation reactions, resulting in lower substrate levels for ester production (Landaud, Latrille and Corrieu 2001).

### Genetic factors and ester production

As acetate esters are quantitatively the most abundant group of esters in industrial fermentations, and are shown to have a major impact on flavor, it is not surprising that researchers have often aimed to hijack the yeast's ester production to diversify the organoleptic characteristics of many diverse fermented foods. The total ester production and the relative proportions of each individual ester differs dramatically between species and strains (Steensels *et al.*2014a; Padilla, Gil and Manzanares 2016). Thus, the most straightforward way to vary ester levels in fermentation is to vary the yeast strain. Metabolic engineering to control ester formation has mostly targeted *ATF1* and *ATF2* expression or activity (Lilly, Lambrechts and Pretorius 2000; Hirosawa *et al.*^2004^; Lilly *et al.*^2006^; Swiegers *et al.*^2006^). Modulating expression of *IAH1*, an esterase, also affects ester concentrations (Lilly *et al.*^2006^; Zhang *et al.*^2012^). Sexual hybridization has also been successfully applied to modulate ester production. Breeding methods have helped increase and diversify ester production of commercial ale (Steensels *et al.*2014a), lager (Mertens *et al.*^2015^), sake (Yoshida *et al.*^1993^; Kurose *et al.*^2000^), wine (Bellon *et al.*^2013^) and even chocolate (Meersman *et al.*^2016^).

Since formation of these compounds does not necessarily impart a fitness advantage, there is no straightforward way to select for desired ester production in experimental evolution, mutagenesis or breedings set ups. Therefore, other approaches have been developed to select for enhanced esters. Growth in the presence of a leucine analog (5,5^΄^,5^″^-trifluor-DL-leucine) selects for variants with reduced positive feedback on leucine production which results in increased production of isoamyl alcohol and isoamyl acetate (Oba *et al.*^2005^). Similarly, growth with phenylalanine analogues (*o-*fluoro- and *p-*fluro-DL-phenylalanine) selects for 2-phenylethyl acetate producers (Fukuda *et al.*^1990^, ^1991^). There have been interesting attempts to selectively enhance variations in either *ATF1* or *ATF2* given the variations in which types of acetate esters are produced. Growth with farnesol analogs (1-farnesylpyridinium) favors Atf1 activity (Hirooka *et al.*^2005^), while supplementing medium with pregnenolone favors Atf2 activity (Tsutsumi *et al.*^2002^; Kitagaki and Kitamoto 2013). In the latter example, the harmful steroid is metabolized by Atf2 and therefore selects for strains with enhancements of Atf2 activity. Those mutants would be able to increase levels of isoamyl acetate without affecting ethyl acetate.

Experimental evolution utilizing lipid synthesis inhibitors has also resulted in strains with enhanced ester production. Selection on aureobasidin, a sphingolipid biosynthesis inhibitor, resulted in mutations in *MGA2* which has been implicated in *ATF1* regulation (Takahashi *et al.*^2017^). Growth on cerlulin, a fatty acid synthesis inhibitor, selected for mutants of *FAS2*, a fatty acid synthetase, with enhanced production of ethyl esters and the additional benefit of reduced acetic acid levels (see Fig. 5) (Ichikawa *et al.*^1991^). A self-cloning sake strain equipped with this mutation became the first genetically modified microorganism approved for industrial use in Japan (Aritomi *et al.*^2004^).

### Physiological and ecological roles of esters

The physiological role of ester production in yeast has been under debate for several decades. It has been hypothesized that ester synthesis helps to tune intracellular redox balance (Malcorps and Dufour 1992) and that some esters help to maintain plasma membrane fluidity under stressful conditions (Mason and Dufour 2000). Additionally, esterification of toxic medium-chain fatty acids may facilitate their removal from cells via diffusion through the plasma membrane (Nordström 1964b). While the intracellular roles are not quite understood, recently it has become clear that esters have significant roles extracellularly.

Of the many volatile compounds produced by yeast, esters represent one of the most important groups that can act as insect semiochemicals, signaling the presence of rotting fruits (El-Sayed *et al.*^2005^). Fruity esters such as isoamyl acetate, ethyl acetate and 2-phenylethyl acetate represent the core attractants of various insects (Davis *et al.*^2013^; Christiaens *et al.*^2014^; Scheidler *et al.*^2015^). Deletion of *ATF1* in *S. cerevisiae* significantly reduces attraction of *Drosophila melanogaster* and simple re-addition of isoamyl acetate or ethyl acetate can restore the flies’ behavior (Christiaens *et al.*^2014^). Isoamyl acetate is also responsible for attraction of *D. simulans*, but the attraction is strongly dependent on the background chemical matrix (Günther *et al.*^2015^). There are also examples of possible species-specific responses to various ester compounds. *Drosophila suzukii* has a significantly higher response to isobutyl and isoamyl acetate, whereas *D. melanogaster* responds to ethyl hexanoate (Keesey, Knaden and Hansson 2015; Scheidler *et al.*^2015^). The herbivorous drosophilid, *Scaptomyza flava*, a relative of *D. melanogaster*, has lost its ability to detect most yeast volatiles (Goldman-Huertas *et al.*^2015^). Genes encoding for neuronal receptors responsible for detecting esters are either deleted or have loss of function mutations in *S. flava*, demonstrating the important connection between yeast volatiles and locating microbial food sources.

The black calla lily (*Arum palaestinum*) has taken advantage of the drosophilids’ ability to detect esters. This plant has evolved to mimic yeast fermentation volatiles specifically by producing 2,3-butanediol acetate and acetoin acetate to lure drosophilids for pollination (Stökl *et al.*^2010^).

Recent evidence indicates that interactions within the *D. melanogaster* microbiome can alter behavior of the fly (Fischer *et al.*^2017^). While the flies feed on yeasts, lactic and acetic acid bacteria are major constituents of its gut microbiome. In fermenting fruits, all three microorganisms co-exist and the growing microbes create a collaborative volatile profile which enhances attraction of *D. melanogaster.* Acetate esters (isobutyl acetate, isoamyl acetate, 2-phenylethyl acetate, 2-methylbutyl acetate, methyl acetate, ethyl acetate) along with acetic acid and acetoin were determined as the key compounds in this interaction (Fischer *et al.*^2017^).

In combination with higher alcohols, esters can be attractive for agricultural pests such as the coffee bean weevil *Araecerus fasciculatus* (Coleoptera: Anthribidae) and *Carpophilus* beetles as they mimic volatiles of fermenting fruits (described in the previous section) (Phelan and Lin 1991; Nout and Bartelt 1998; Yang *et al.*^2016^). Codling moths *Cydia pomonella*, a common apple pest, utilizes esters and other aroma compounds emitted by *Metchnikowia* yeasts to locate suitable ovipositioning sites (Witzgall *et al.*^2012^).

In addition to insects, the earthworm *Eisenia fetida* uses volatile cues, such as ethyl pentanoate and ethyl hexanoate, to navigate towards its food source *Geotrichum candidum*, a yeast-like mold frequently used in the dairy industry (Zirbes *et al.*^2011^). Additionally, esters emitted by *S. cerevisiae*, such as methyl acetate, ethyl acetate, propyl acetate, butyl acetate and amyl acetate, have strong attractive effects on nematode worms (Balanova *et al.*^1979^).

Yeast-produced esters can also mediate host–parasite interactions. Honey bees produce isoamyl acetate-containing alarm pheromones that defend the hive against several predators and parasites. The beetle *Aethina tumida* (Coleoptera: Nitidulidae) is attracted to the isoamyl acetate. The beetles can vector the yeast *Koamaea ohmeri* to the hive which then begins to ferment and produce more isoamyl acetate in high concentrations. This amplifies the attraction of beetles and results in a vast infestation of beetles and larvae, causing enormous damage to the hive (Torto *et al.*^2007^). Similarly, the parasitic wasp *Leptopilina heterotoma* is attracted to ethyl acetate (along with ethanol and acetaldehyde) which puts it in proximity to its potential host, *D. melanogaster* (Dicke *et al.*^1984^).

Similar to the higher alcohols, esters can have antifungal effects, possibly to eliminate competition for the yeasts producing them. *Pichia anomala*, *P. kluyveri* or *Hanseniaspora uvarum* all secrete 2-phenylethyl acetate which can strongly inhibit growth and mycotoxin production by the fungus *Aspergillus ochraceus* (Masoud, Poll and Jakobsen 2005).

## AMINO ACID METABOLITES: SULFUR COMPOUNDS

The generic classification of ‘sulfur-containing’ opens a large and diverse array of compounds to consider including everything from basic thiols (such as hydrogen sulfide or methanethiol) and sulfides (dimethyl sulfide, dimethyl disulfide, etc.), thioethers and thioesters, sulfur-containing aldehydes and alcohols, as well as larger, polyfunctional thiols. Given the extensive list of potential compounds, we focus on the assimilation of sulfur, the connections to amino acid metabolism and industrially relevant sulfur compounds.

### Biochemistry of sulfur assimilation and metabolism

**Figure 6.**
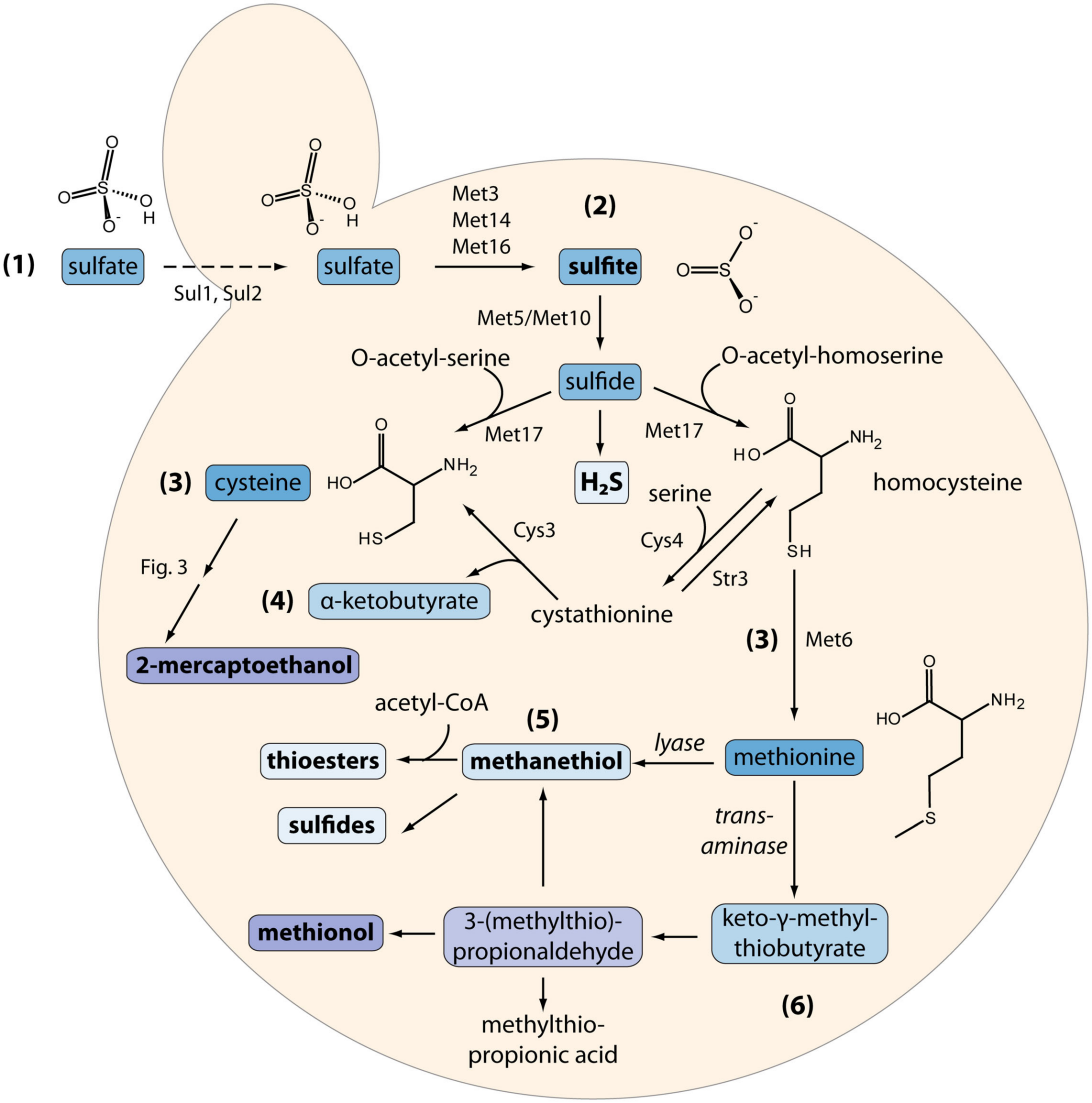
**Sulfate reduction pathway leading to the production of sulfur-containing amino acids and compounds.** (1) Extracellular sulfate is taken up through two transporters, Sul1 and Sul2, and sequentially reduced to sulfite and sulfide. (2) Excess sulfide can be converted to hydrogen sulfide which diffuses out of the cell or (3) assimilated into amino acid synthesis pathways. (4) Production of α-ketobutyrate links this pathway to threonine and the branched amino acid synthesis pathways (Fig. 2). (5) Methionine can be acted on by a lyase to form methanethiol, which is a major precursor for numerous sulfur-containing aroma compounds. (6) Methanethiol can also be produced via transamination of methionine, which is also the first step of the Ehrlich pathway (Fig. 3). Adapted from Landaud (2008), Pereira *et al*. (2008), and Saccharomyces Genome Database (Cherry *et al.*^2012^).

All yeast-produced sulfur compounds arise during the catabolism or anabolism of the sulfur-containing amino acids methionine and cysteine. Since these amino acids are found at relatively low concentrations in both natural and industrial environments, yeasts are required to assimilate inorganic sulfur via the sulfate reduction sequence (Fig. 6). Sulfates are sequentially reduced to sulfide which can combine with a nitrogen source (O-acetyl-serine or O-acetyl-homoserine) to form cysteine and subsequently, methionine. From this point, the amino acids can be incorporated into protein or re-metabolized to form other volatile sulfur compounds. In cases of low nitrogen, the amount of available O-acetyl-serine or O-acetyl-homoserine is limited, and there is an overproduction of sulfide. This is converted to hydrogen sulfide to allow for diffusion out of the cell (Jiranek, Langridge and Henschke 1995; Spiropoulos *et al.*^2000^; Mendes-Ferreira, Mendes-Faia and Leão 2002; Swiegers and Pretorius 2005). Additionally, it has recently been shown that some sulfur compounds, such as ethanethiol, S-ethyl thioacetate and diethyl disulfide, can be synthesized from excess H_2_S, independent of methionine synthesis (Kinzurik *et al.*^2016^).

From newly synthesized or exogenously added methionine and cysteine, all other volatile sulfur compounds can be produced. Some of these pathways have not been fully mapped in *S. cerevisiae*, but a general scheme can be drawn based on studies done on sulfur pathways in bacteria and other yeast species (Fig. 6). Bacteria have been more widely studied in regard to sulfur production since the negative odors are generally associated with spoilage or desired aromas in specific types of cheese which utilize lactic acid bacteria (Kieronczyk *et al.*^2003^). Tracing studies and genetic engineering attempts to manipulate levels of H_2_S and the more desirable sulfur compounds have provided insight into potential biosynthetic pathways (Arfi, Landaud and Bonnarme 2006; Cordente *et al.*^2012^).

Cysteine and methionine breakdown has been linked to dimethyl sulfide (DMS) production but it can also be formed from the reduction of dimethyl sulfoxide (DMSO) by Mxr1 (methionine sulfoxide reductase) (Hansen 1999). For most other sulfur-containing compounds, methanethiol is considered the primary precursor. Two different pathways lead to the production of methanethiol: the lyase pathway or the transamination pathway (Fig. 6, step 5). Demethiolation of methionine by a lyase is more comprehensively understood in bacteria but it does occur in yeasts (Landaud, Helinck and Bonnarme 2008). The transamination pathway is essentially the Ehrlich pathway. The intermediate keto-γ-methylthiobutyrate (also referred to as 4-methylthio-2-oxobutyric acid or MOBA) can undergo a variety of chemical and enzymatic reactions including conversion to methanethiol. If MOBA continues via the Ehrlich pathway, there is subsequent production of methional, then methionol (via reduction) or methylthio-propionic acid (via oxidation). Cysteine can also undergo conversion to the respective higher alcohol, 2-mercaptoethanol. Methanethiol can be produced through oxidation or acylation reactions (Landaud, Helinck and Bonnarme 2008).

There is an important category of sulfur-containing compounds that are not directly synthesized by yeast. Polyfunctional thiols are present in the biomass used for fermentation but as non-volatile precursors. The cystathionine lyases Cys3, Irc7 and Str3 release the polyfunctional thiols from the cysteine conjugates (Tominaga *et al.*^1998^; Howell *et al.*^2005^; Holt *et al.*^2011^; Roncoroni *et al.*^2011^).

### Sulfur compounds in industry

Sulfur compounds are most relevant in beer, wine and cheese-making industries. Unlike fusel alcohols or esters, some sulfur compounds are classified as positive while others are considered negative odors. For example, the classic ‘rotten-egg’ odor usually associated with sulfur comes from hydrogen sulfide (H_2_S) while furfurylthiol smells of roasted coffee. Other negative sulfur compounds include methanethiol (cooked cabbage), sulfides (cabbage, cauliflower, garlic) and methylthioesters (cheesy, chives) (Cordente *et al.*^2012^). Interestingly, the perception of these compounds is highly context specific. While DMS typically smells of cabbage, it can convey desired aroma notes to lager beers and whiskey (Anness and Bamforth 1982; Hansen *et al.*^2002^). Similarly, some of the sulfur-containing aromas are produced by yeasts on the surface of soft cheeses and contribute to their distinctive odor (Landaud, Helinck and Bonnarme 2008).

Some aroma-enhancing volatile thiols are produced by wine yeast from precursors present in grape must. Of interest are 4-mercapto-4-methylpentan-2-one (4MMP), 3-mercaptohexan-1-ol (3MH) and 3-mercaptohexyl acetate (3MHA), which impart box tree (4MMP), passionfruit, grapefruit, gooseberry and guava aromas (3MH and 3MHA) on the wine (Tominaga *et al.*^1998^; Dubourdieu *et al.*^2006^).

Sulfites can act as an antioxidant in wine and beer as well as protect against bacterial and *Brettanomyces* spoilage (Suzzi, Romano and Zambonelli 1985; Divol, Toit and Duckitt 2012). However, sulfites produced by yeast are at relatively low levels since they are reduced to be incorporated into amino acids. Therefore, these are sometimes added prior to bottling to help stabilize the final product.

### Environmental parameters and sulfur compound production

Since several sulfur compounds are considered to negatively affect product quality, several strategies have been developed to reduce their emission (Table 7). Low nitrogen conditions increase the yeast cell's need for amino acids which would increase general sulfur assimilation. This leads to increased production of H_2_S so it has been common practice for decades to add nitrogen sources to fermentation medium (Jiranek, Langridge and Henschke 1995; Mendes-Ferreira, Mendes-Faia and Leão 2004). However, this effect is dependent on the timing of supplementation, yeast strain and the presence of methionine (Spiropoulos *et al.*^2000^; Mendes-Ferreira *et al.*^2010^; Barbosa, Mendes-Faia and Mendes-Ferreira 2012). The strongest decrease in H_2_S levels is obtained when nitrogen source is added concurrently with methionine.

**Table 7.** Effect of environmental parameters on sulfur compound production.

**Parameter**	**Condition**	**Effect on sulfur compound production**	**Reference**
**Temperature**	Increase	Increase (thiols)	Howell *et al.* (2004), Masneuf-Pomarede *et al.* (2006)
**pH**	Decrease	Decrease (H_2_S, methanethiol, DMS)	Bekker *et al.* (2016)
**Oxygen (fermentation)**	Increase	Decrease (H_2_S, methanethiol, ethanethiol, methylthioacetate, ethylthioacetate, DMS)	Bekker *et al.* (2015)
**Oxygen (post-bottling)**	Increase	Decrease (H_2_S, methanethiol)	Ugliano *et al.* (2012)
**Medium composition**			
***Copper sulfate***	Supplementation	Decrease (H_2_S and thiols; oxidation)	Kreitman *et al.* (2016)
***N source (total)***	Increase	Decrease (H_2_S; dependent on timing and methionine concentration)	Mendes-Ferreira *et al.* (2010), Spiropoulos *et al.* (2000)
***Botrytis cinerea* infection**	Increase	Increase (thiols)	Thibon *et al.* (2010)

Perhaps one of the most common problems in the wine industry is finding a balance between limiting production of the undesirable H_2_S while increasing levels of aroma-enhancing volatile thiols. Complete wine fermentations are sometimes treated with copper sulfate, a process referred to as copper fining, which effectively removes H_2_S (Clark, Wilkes and Scollary 2015). However, the copper only requires presence of a free thiol group to form a stable complex and will therefore also decrease levels of desirable thiol compounds. Furthermore, this strategy is ineffective in removing several sulfuric off-odors that lack a free thiol group, such as disulfides, thioacetates and cyclic sulfur (Kreitman *et al.*^2016^).

Oxygenation both during fermentation or post-bottling can also influence volatile sulfur compound profiles in wine. Oxygen treatment during fermentation can reduce concentrations of H_2_S, methanethiol and ethanethiol (Bekker *et al.*^2015^). The effect of exposure after bottling is dependent on oxygen ingress through the bottle cap or cork. More porous closures allow for some gas exchange and are correlated with lower H_2_S and methanethiol levels (Ugliano *et al.*^2012^). DMS and DMDS levels are unaffected; however, desirable volatile thiols are also reduced and are thus better conserved in air-tight conditions compared to oxygen permeable conditions (Lopes *et al.*^2009^).

### Genetic factors and sulfur compound production

Sulfur compound production widely varies between *S. cerevisiae* strains and other species. Genetic engineering strategies have targeted several of the genes associated with sulfur assimilation (Fig. 6). Mutation of *MET5* or *MET10* blocks the conversion of sulfite to sulfide and reduces H_2_S production (Sutherland *et al.*^2003^; Cordente *et al.*^2009^; Linderholm *et al.*^2010^; Bisson, Linderholm and Dietzel 2013). Overexpression of the cystathionine synthetase *CYS4* also reduces H_2_S production by driving the sulfide towards amino acid synthesis (Tezuka *et al.*^1992^). Mutating *MET14* limits sulfur assimilation overall (Donalies and Stahl 2002). Additionally, mutations in *MET2* (produces O-acetyl-homoserine) or *SKP2* (a potential regulator of sulfur assimilation genes) increase levels of sulfite and H_2_S (Hansen and Kielland-Brandt 1996; Yoshida *et al.*^2011^). DMS levels can be reduced by disrupting *MXR1*, which prevents the conversion of DMSO to DMS (Hansen *et al.*^2002^).

Enhanced release of aromatic thiols from biomass precursors can be achieved by variations in the lyases, specifically the β-lyases *IRC7* and *STR3*. Many *S. cervisiae* strains have 38 bp deletion in the *IRC7* gene, resulting in low levels of 4MMP. Strain selection for β-lyase activity or overexpressing *STR3* or a full-length copy of *IRC7* greatly enhances 4MMP and 3MH release (Holt *et al.*^2011^; Roncoroni *et al.*^2011^; Belda *et al.*^2016^).

### Physiological and ecological roles of sulfur compounds

Hydrogen sulfide plays an important role in the physiology of yeast cells. As described above in the acetaldehyde section, yeast cells exhibit glycolytic oscillations, in which they coordinate their metabolism. Hydrogen sulfide can also cause respiration inhibition and therefore plays a role in regulating respiratory oscillations (Sohn, Murray and Kuriyama 2000; Lloyd and Murray 2006).

Methionol has been shown to activate an olfactory response neuron in *D. melanogaster* (de Bruyne, Foster and Carlson 2001) and attract the fruit flies (Farhadian *et al.*^2012^; Knaden *et al.*^2012^) but concentrations used in those studies were higher than what is typically produced by fermenting yeasts. However, it has been shown that natural levels of methionol from vinegar and wine elicit an antennal response from *D. suzukii* and when mixed with other compounds (acetic acid, acetoin and ethanol) it effectively attracts the flies (Cha *et al.*^2014^). This indicates that methionol could play a relevant ecological role in yeast–drosophilid communication.

Truffles host various yeast and bacteria and while the production of volatile compounds overlaps between the species, it has been speculated that yeasts contribute to the truffle aroma, largely defined by sulfuric compounds such as DMS, DMTS and 3-(methylsulfanyl)-propanal (Buzzini *et al.*^2005^; Vahdatzadeh, Deveau and Splivallo 2015). DMS is one of the defining cues for pigs, which use truffles as a food source, as well as for dogs, which are trained by humans to locate underground truffles (Talou *et al.*^1990^).

## PHENOLIC COMPOUNDS

### Biochemistry of phenolic compound production

Pre-treatment of various lignin polymers of plant cell walls is a common practice in the fuel and beverage industries. The bioprocessing of these polymers prior to the fermentation process releases a variety of furans, carboxylic acids and phenolic compounds which can greatly inhibit microbial growth (Klinke, Thomsen and Ahring 2004). Many microbial species, such as *Saccharomyces cerevisiae, Aspergillus niger, Pseudomonas aeruginosa* and *Escherichia coli*, counteract the negative impact by converting these compounds into less toxic molecules. For example, vanillin, a phenolic guaiacol, can be detoxified by conversion to vanillyl alcohol by yeast Adh6 (Wang *et al.*2016a). Several of the hydroxycinnamic acids, such as cinnamic acid (phenylacrylic acid), caffeic acid, ferulic acid and *p*-coumaric acid, can be decarboxylated to less toxic phenolic compounds which have a large impact on industrial fermentations (Fig. 6).

In *S. cerevisiae*, there are two enzymes essential for decarboxylation of the hydroxycinnamic acids encoded by *PAD1* and *FDC1* (phenylacrylic acid decarboxylase and ferulic acid decarboxylase). For several years, it was unclear how the genes interacted to produce phenolic compounds. In some studies, *PAD1* was assumed to be the sole responsible enzyme for this reaction as deletion or mutation resulted in complete loss of activity but it was clearly demonstrated that both *PAD1* and *FDC1* are required for the decarboxylation of hydroxycinnamic acids (Mukai *et al.*^2010^). It has now been shown that *PAD1* possesses no decarboxylase activity but instead is responsible for formation of a modified flavin mononucleotide (FMN) which is required for *FDC1* decarboxylase activity (Lin *et al.*2015a; Payne *et al.*^2015^; White *et al.*^2015^).

### Phenolic compounds in industry

**Figure 7.**
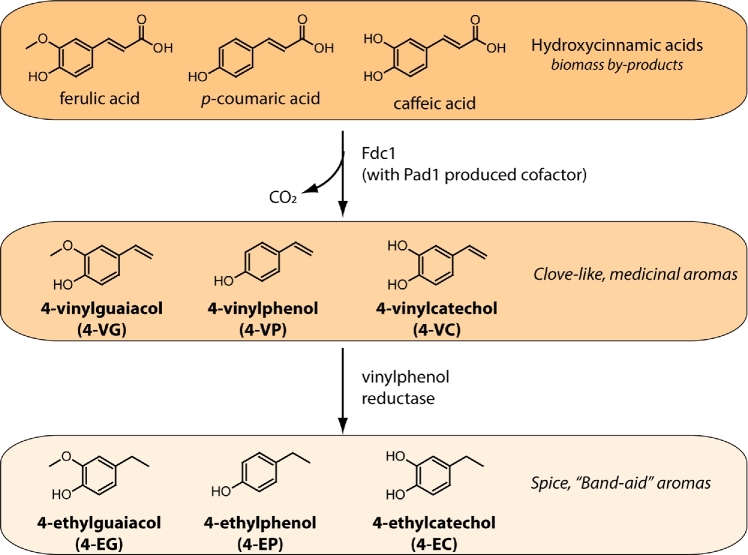
**Production of phenolic compounds**. Hydroxycinnamic acids are released during pre-processing of biomass. Yeast cells can decarboxylate these toxic compounds to less harmful forms through the actions of Fdc1. Fdc1 requires a cofactor FMN which is produced by Pad1. The compounds are then secreted and can be further reduced by a vinylphenol reductase, typically by contaminating yeast or bacterial species.

During fermentation, the actions of Pad1 and Fdc1 convert ferulic acid, *p-*coumaric and caffeic acid to 4-vinylguaiacol (4-VG), 4-vinylphenol (4-VP) and 4-vinylcatechol (4-VC), respectively (Fig. 7). Subsequently, these compounds can be reduced to form 4-ethylguaiacol (4-EG), 4-ethylphenol (4-EP) and 4-ethylcatechol (4-VC) by vinylphenol reductase (Vanderhaegen *et al.*^2003^; Vanbeneden, Delvaux and Delvaux 2006; Hixson *et al.*^2012^). Both 4-VG and 4-EG are associated with more pleasant clove-like or spicy aromas, while 4-VP and 4-EP aromas are considered more medicinal and ‘Band-Aid’-like. As *Saccharomyces* generally lacks reductase activity, 4-EG, 4-EP production during fermentation is an indicator of the presence of *Brettanomyces* (Steensels *et al.*^2015^). These phenolic compounds are significant contributors to fermentation aromas but their role is ambiguous. In certain specialty beer styles, such as wheat, Hefeweizen, Lambic, American coolship ale and acidic ale beer, the phenolic flavors are desired and help define the style. However, the same compounds are perceived negatively in most other fermented beverages and are commonly referred to as ‘phenolic off-flavors’ (POF) (Vanbeneden 2007).

### Environmental parameters and phenolic compound production

Given the general association as ‘off-flavors’, several aspects of the fermentation process have been modified to reduce phenolic compound production (Table 8). The undesired presence of *Brettanomyces* during fermentation can be attenuated by various inhibitors (e.g. sulfites or chitosans) or electric currents. Production of phenolic compounds by *Saccharomyces* heavily depends on the precursor availability in the fermentation medium. Increased precursor concentrations not only increase substrate availability but also activate transcription of *PAD1* and *FDC1* (Vanbeneden 2007). Other fermentation parameters, such as temperature and carbon source, have been shown to affect formation of phenolic compounds, but the underlying mechanisms are not understood (Vanbeneden 2007; Cui *et al.*^2015^).

**Table 8.** Effect of environmental parameters on phenolic compound production.

**Parameter**	**Condition**	**Effect on phenolic compound production**	**Reference**
***Brettanomyces***			
**Medium composition**			
***Antimicrobial agents***			
***(sulfite, chitosans, …)***	Supplementation	Decrease (inhibits growth)	Portugal *et al.* (2014)
***Weak acids and sorbic acid***	Supplementation	Decrease (inhibits growth)	Wedral *et al.* (2010)
**Low electric current**	Application of ∼200 mA	Decrease (inhibits growth)	Zuehlke *et al.* (2013)
**Pulsed electric field**	Application of ∼30 kV/cm, 1–4 μs pulses	Decrease (inhibits growth)	Zuehlke *et al.* (2013)
***Saccharomyces***			
**Temperature**	Increase	Increase	Vanbeneden (2007), Cui *et al.* (2015)
**Medium composition**			
***C source***	Glucose	Increase (compared to fructose, maltose, sucrose, galactose)	Vanbeneden (2007)
***C source***	Fructose, maltose, sucrose	Increase (compared to galactose); decrease (compared to glucose)	Vanbeneden (2007)
***C source***	Galactose	Decrease (compared to glucose, fructose, maltose, sucrose)	Vanbeneden (2007)
**Top pressure**	Increase	Decrease (increase in dissolved CO_2_)	Vanbeneden (2007)
**Fermentation practice**	Top cropping	Decrease (less yeast sedimentation)	Vanbeneden (2007)

### Genetic factors and phenolic compound production

Surprisingly few attempts have been performed to modify phenolic compound production in industrial strains. This is due in part to the simplicity of their production and the fact that many industrial yeasts have already acquired natural mutations to block phenolic compound production. It has recently been established that selection for *PAD1* and *FDC1* loss-of-function mutants is one of the key drivers in the domestication of industrial *S. cerevisiae* lineages associated with beer and sake production (Gallone *et al.*^2016^; Gonçalves *et al.*^2016^). This selection is not observed in baking or bioethanol strains as in these cases, phenolic compounds are likely less detrimental, either because the flavor disappears during baking or the product is not destined for consumption. Additionally, for strains used in beer styles where phenolic compounds are desired, selection for mutations in these genes is not observed.

### Physiological and ecological roles of phenolic compounds

The POF-negative character of many industrial yeasts is especially striking since the phenotype is preserved in all wild strains that have currently been analyzed, which indicates a strong fitness advantage of these genes in natural environments (Gallone *et al.*^2016^). Since hydroxycinnamic acids are antimicrobial compounds, the ability of some yeasts to convert these acids to less harmful phenolic compounds provide them with resistance and promotes growth (Baranowski *et al.*^1980^; Larsson, Nilvebrant and Jönsson 2001; Richard, Viljanen and Penttilä 2015). Additionally, formation of the ethyl derivatives could play a role in maintaining redox balance in the cell in oxygen-limited conditions. Low oxygen enhances activity of the vinylphenol reductase (Fig. 7) and subsequently reduces levels of its cofactor, NADH (Curtin *et al.*^2013^).

*Drosophila melanogaster* uses volatile ethyl phenols as indicators for the presence of hydroxycinnamic acids which are potent dietary antioxidants. Since the insects do not possess the ability to detect the acids directly, they have developed specialized olfactory neurons for detecting the ethyl phenols instead (Dweck *et al.*^2015^).

## CONCLUSION

Humans realized the potential of fermentation several thousand years ago, and have since been exploiting the natural versatility of yeast aroma production. Fermented foods and beverages provide several advantages including longer shelf lives and a pleasing euphoric effect. Over time, the procedures for fermentations became more sophisticated and more refined. Eventually, other uses for fermentation became apparent and the use of yeast for industrial purposes sparked a whole new field of research and development. There is now genetic evidence that demonstrates how much humans have driven the evolution of industrial yeast species to select for desired aroma traits (Gallone *et al.*^2016^, Gonçalves *et al.*^2016^). Moreover, in the past few decades, new technologies have significantly advanced and refined the selection process (Steensels *et al.*2014b). We now utilize specific yeast strains to produce biofuels, pharmaceutical compounds, flavors and fragrant additives.

Selection for specific aromas has also been observed in natural strains (Gallone *et al.*^2016^) but in some cases, wild yeasts maintain some aromas that humans have deemed undesirable. There are also species-specific enhancements of various aroma compounds through small variations in the biosynthetic genes. This leads to questions about what possible physiological roles the different aroma compounds may have and whether there are fitness advantages to produce them.

Microbial aroma compound production is important in both industrial and ecological settings. Aroma compounds very often signal desirability or identify potentially harmful conditions. In many cases, the physiological role of aroma formation remains unknown, but several hypotheses have been proposed. Some aromas are simply by-products of detoxification of otherwise harmful compounds, such as the conversion of hydroxycinnamic acids and esterification of toxic medium chain fatty acids (Nordström 1964b, Klinke, Thomsen and Ahring 2004). Similarly, the vicinal diketones are an indirect result of secreting compounds that otherwise can cause stress on the cell (van Bergen *et al.*^2016^). Moreover, compounds such as acetaldehyde can coordinate physiological oscillations between neighboring cells and provides a larger ecological context for aroma production (Richard *et al.*^1996^).

**Figure 8.**
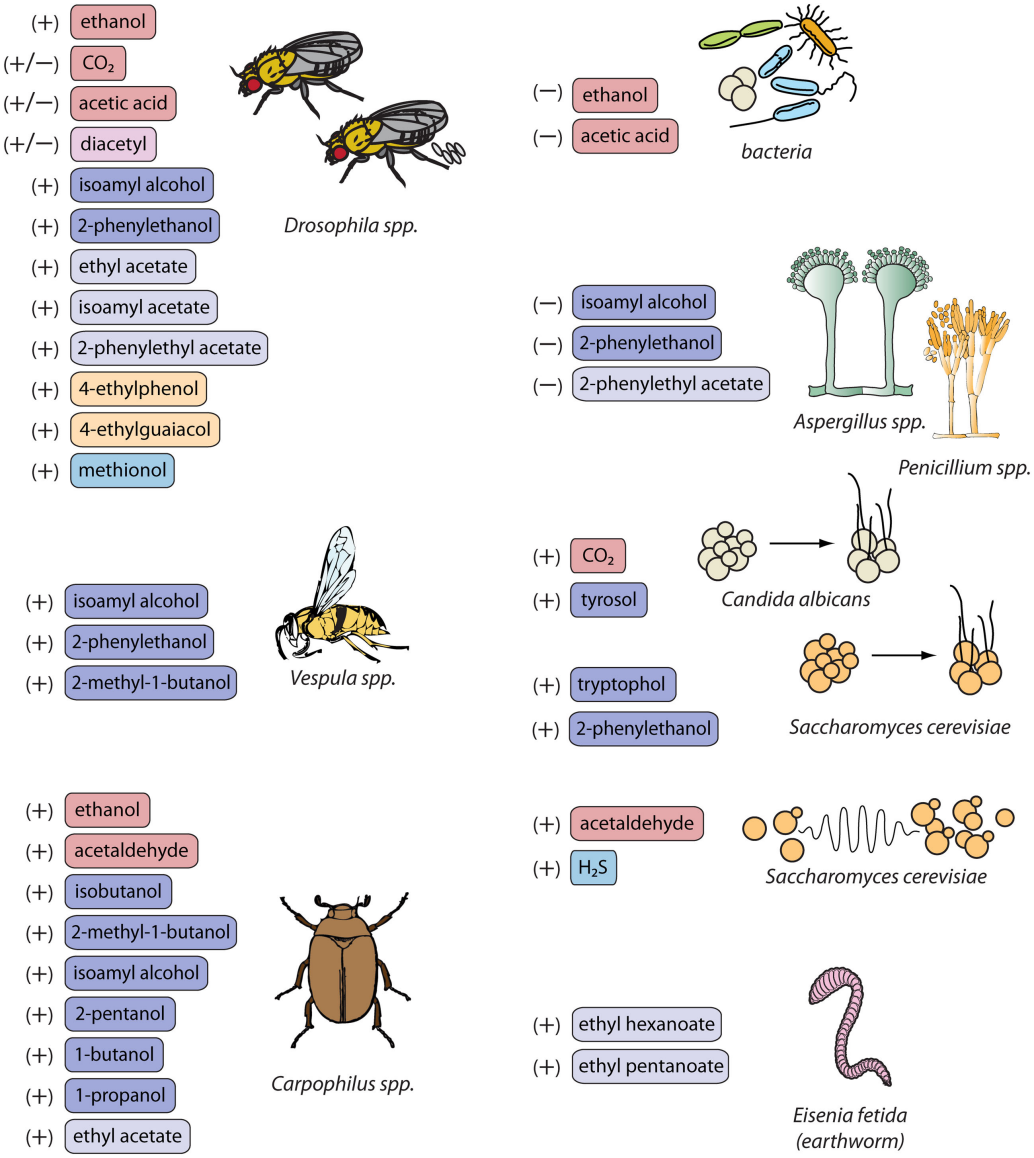
**Summary of the ecological roles of aroma compounds**. This review has summarized a variety of physiological and ecological roles of yeast aroma compounds. This figure depicts some of the major organisms described to illustrate the vast number of compounds that they interact with. Positive (+) indicates a generally positive interaction such as attraction, increased growth or behavior. Negative (–) indicates a negative interaction such as inhibited growth or repulsion.

Yeasts inhabit a large array of ecological niches, from the guts of insects to the fruits and nectar of various plant species. Emissions from microorganisms may signal aspects of food and habitat suitability and therefore attract or repel foraging insects (Fig. 8). Yeasts associated with insects are known to provide nutrition for the insects so it would seem counterintuitive for yeasts to enhance attraction for potential predators. However, recent work has shown that production of insect-attracting volatiles by yeasts represents a clever strategy to travel to new environments, and thus enhances the dispersal and survival of otherwise non-motile yeasts (Davis, Boundy-Mills and Landolt 2012, Christiaens *et al.*^2014^).

While there are many examples of yeast aromas affecting the behavior of insects, our understanding of the interactions between yeast and their vectors using volatile cues is still far from complete. The perception of many yeast aroma compounds is strongly dependent on synergistic effects between compounds. This is observed in industry where individual compounds can be masked or highlighted when combined with other compounds. This type of synergism is even more clearly manifested in nature, where most yeast volatile compounds elicit stronger behavioral responses when presented in blends or with a relevant background chemical context (Davis and Landolt 2013; Günther *et al.*^2015^). Though we know quite a lot about individual aroma compounds, the complex interactions between them are relatively understudied. Additionally, it is likely that there are more aroma compounds to be identified, especially in an ecological context. Moreover, it is yet unclear if the insect and animal recipients perceive the compounds discretely or as a blend. Such interactions could also be interesting from a human perspective especially in the case of bioremediation in agriculture, where microbial-produced compounds can be exploited as insect repellants or attractants. The plethora of already observed interactions that are influenced by aroma compounds illustrates that aroma-producing microbes may play important, yet underestimated roles in the ecosystem.
